# Developmental dynamics of marmoset prefrontal cortical SST and PV interneuron networks highlight primate-specific features

**DOI:** 10.1242/dev.204254

**Published:** 2025-05-19

**Authors:** Nafiseh S. Hosseini Fin, Adrian Yip, Jack T. Scott, Leon Teo, Jihane Homman-Ludiye, James A. Bourne

**Affiliations:** ^1^ Australian Regenerative Medicine Institute, 15 Innovation Walk, Monash University, Clayton, VIC 3800, Australia; ^2^ Section on Cellular and Cognitive Neurodevelopment, Systems Neurodevelopment Laboratory, National Institute of Mental Health, Bethesda, MD 20892, USA; ^3^ Monash MicroImaging, 15 Innovation Walk, Monash University, Clayton, VIC 3800, Australia

**Keywords:** Ion channels, Maturation, Primate, Dorsolateral prefrontal cortex, Neurodevelopmental disorders

## Abstract

The primate prefrontal cortex (PFC) undergoes protracted postnatal development, crucial for the emergence of cognitive control and executive function. Central to this maturation are inhibitory interneurons (INs), particularly parvalbumin-expressing (PV^+^) and somatostatin-expressing (SST^+^) subtypes, which regulate cortical circuit timing and plasticity. While rodent models have provided foundational insights into IN development, the trajectory of postmigratory maturation in primates remains largely uncharted. In this study, we characterized the expression of PV, SST, the chloride transporter KCC2, and the ion channels Kv3.1b and Nav1.1 across six PFC regions (areas 8aD, 8aV, 9, 46, 11 and 47L) in the postnatal marmoset. We report a prolonged maturation of PV^+^ INs into adolescence, accompanied by progressive upregulation of ion channels that support high-frequency firing. In contrast, SST^+^ INs show a postnatal decline in density, diverging from rodent developmental patterns. These findings reveal distinct, cell type-specific maturation dynamics in the primate PFC and offer a developmental framework for understanding how inhibitory circuit refinement may underlie vulnerability to neurodevelopmental disorders.

## Teaser

**Summary:** Identification of prolonged PV interneuron maturation and distinct SST expression patterns in early postnatal and adult marmoset brain offers new insights into primate prefrontal cortex development, highlighting differences from rodent cortical maturation.

## INTRODUCTION

Distinguishing the primate, including human, prefrontal cortex (PFC) from rodent PFC is the presence of a distinct granular layer (Roberts and Clarke, 2019; Watakabe et al., 2023), which orchestrates specific functions and connectivity (Preuss and Wise, 2022). An evolved thalamic network, the medial pulvinar (PM), interconnects with these areas (Homman-Ludiye and Bourne, 2019). The dorsolateral prefrontal cortex (DLPFC) in anthropoids includes Brodmann areas (BA) 8, 9 and 46, receiving input from multiple sensory and multimodal areas, and projecting to occipital, temporal and parietal lobes, and sensorimotor cortices. All PFC areas are interconnected through the primate-specific PM nucleus of the thalamus (Homman-Ludiye et al., 2020). The primate PFC has evolved with more discrete areas, from 26 in marmosets to 35 in macaques, and 45 in humans (Watakabe et al., 2023).

Primate brain evolution has also incorporated novel cell types in existing regions (Krienen et al., 2020). The increased neuronal diversity, particularly interneurons (INs), plays a crucial role in primate cognition. While INs account for 15-20% of cortical neurons in rodents, this ratio is 25-34% in primates, accompanied by evolutionary diversification enabling numerous functions (Krienen et al., 2020; Džaja et al., 2014). Understanding the establishment and physiology of primate-specific INs offers insights into primate cognitive functions.

All gamma-aminobutyric acid (GABA)ergic INs in adult primates originate from the embryonic ganglionic eminences (GEs) in the subpallium and the proliferative zones of the dorsal telencephalon (Petanjek et al., 2009; Ma et al., 2013). The medial GE (MGE) produces two major IN classes expressing somatostatin (SST) or parvalbumin (PV) (Fogarty et al., 2007; Kessaris et al., 2014; Wonders and Anderson, 2006). SST^+^ INs are generated early from asymmetrically dividing progenitors in the ventricular zone (VZ) (Bandler et al., 2017; Petros et al., 2015), whereas PV^+^ INs are generated later from MGE subventricular zone (SVZ) progenitors dividing symmetrically (Petros et al., 2015). These subtypes perform distinct functions, such as PV^+^ INs controlling pyramidal cell output, crucial for cognitive development (Miyamae et al., 2017), and SST^+^ INs establishing thalamic inputs during early development (Scott and Bourne, 2022).

As INs integrate into the cortical network, they express subtype markers such as calbindin (CB), PV or SST, and specific ion channels and transporters, including the potassium chloride co-transporter KCC2 (encoded by *SLC12A5*), sodium channel subtype Nav1.1 (encoded by *SCN1A*) and potassium channel subtype Kv3.1b (isoform encoded by *KCNC1*), essential for their functionality (Su et al., 2020; Hendrickson et al., 1991; Virtanen et al., 2021; Ogiwara et al., 2007; Bartholome et al., 2020; Okaty et al., 2009). These proteins play roles in IN homeostasis, calcium buffering, and the transition from excitatory to inhibitory responses (Gu et al., 2018; Lee et al., 2000; Herrmann et al., 2022). The mature network is consolidated by an elaborate scaffold of proteoglycans, collectively known as perineuronal nets (PNNs), which form the extracellular matrix, and myelination (Christensen et al., 2021), marking the closure of maturation and the onset of a functional adult network.

Maturation is a progressive phenomenon that occurs over a protracted period, especially in primates, from infancy for primary sensory areas to adolescence and adulthood for the PFC, as determined by longitudinal magnetic resonance imaging (MRI) analysis (Quah et al., 2022). Utilizing the temporal expression of neurofilament proteins (nonphosphorylated neurofilament; NNF), restricted to a subset of mature excitatory neurons, we previously reported that the maturation of sensory networks was sequential and followed the processing hierarchy, with the primary visual cortex (V1) maturing first, followed by association cortices (Mundinano et al., 2015). A similar approach to studying NNF in the PFC revealed a posterior-to-anterior maturation gradient, with the most anterior areas of the frontal pole developing last (Burman et al., 2007).

Abnormal IN maturation or dysfunction in the PFC is linked to neurodevelopmental disorders such as schizophrenia (SCZ), autism spectrum disorders (ASDs) and attention deficit hyperactivity disorder (ADHD) (Paterno et al., 2020). Disrupting the balance between excitatory and inhibitory activity in the PFC can lead to cognitive impairments and altered behavior (Liu et al., 2021). Understanding the molecular pathways involved in IN maturation is crucial.

Owing to its close phylogenetic relationship to humans and similar cortical organization, the marmoset monkey (*Callithrix jacchus*) serves as a valuable model for studying the development of PV^+^ and SST^+^ INs in the PFC (Homman-Ludiye and Bourne, 2020). Understanding the ontogeny of these INs may reveal conserved mechanisms of cortical circuit formation relevant to human neurodevelopmental disorders.

This study examines the maturation of PV^+^ and SST^+^ INs across marmoset PFC areas 8aD, 8aV, 46, 9, 11 and 47L, which collectively span the anteroposterior and mediolateral PFC axes (Paxinos et al., 2012). We profiled PV, SST, KCC2, Nav1.1, Kv3.1b and PNNs from early postnatal stages to adulthood to define the spatiotemporal and molecular features of IN maturation and their potential implications for neurodevelopmental pathology.

## RESULTS

### Demarcation of areas 8aD, 8aV, 47L, 9, 46 and 11 in the marmoset PFC from infant to adult

**Fig. 1.**
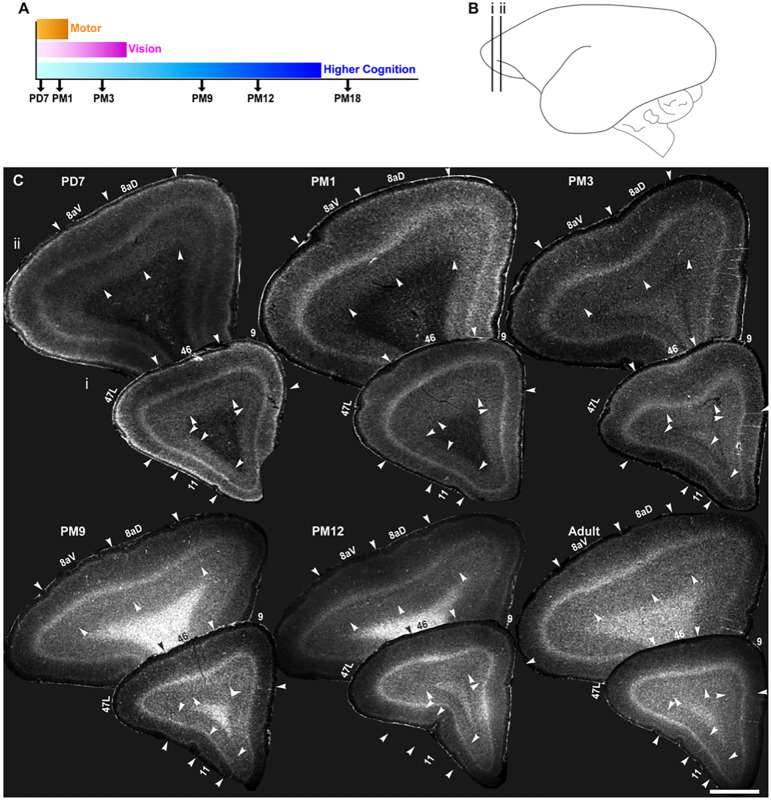
**Cytoarchitecture of marmoset prefrontal neocortical areas 8aD, 8aV, 47L, 11, 9 and 46 from PD7 to adult.** (A) Representation of the developmental stages used in this study: postnatal day (PD) 7, postnatal month (PM) 1, 3, 9, 12 and 18 presented in correlation with the indicative timing of the maturation of the cortical networks sustaining vision and higher cognitive and motor functions. (B) Position of the areas of interest represented on a schematic of the adult marmoset brain modified, with permission, from the marmoset atlas (Paxinos et al., 2012). (C) Representative examples of the laminar cytoarchitecture of the marmoset PFC coronal section stained with Hoechst from birth to adulthood. Changes in cortical layer thickness and density enable the demarcation of the borders between adjacent cortical areas, as indicated by the arrowheads. All the analyzed images were captured in the core region of the areas of interest for consistency within and across stages. Image stitching was performed to generate the low-magnification micrographs of individual cortical sections. Scale bar: 2 mm.

The adult marmoset neocortex has been extensively mapped (e.g. Paxinos et al., 2012). This study extends segmentation to five postnatal stages (Fig. 1A). Observations began on postnatal day (PD) 7 when primary sensorimotor cortices express NNF, marking pyramidal neuron maturation (Bourne and Rosa, 2006). NNF deposition in the basal and apical dendrites of the large pyramidal neurons consolidates the dendritic morphology (Ulfig et al., 1998). Prefrontal areas show minimal NNF expression at PD7, with faint expression in areas 6D and 8aV in L5 (Burman et al., 2007). By 1 month postnatal (PM1), NNF expression remains faint in the PFC but expands to secondary and tertiary sensory areas (Bourne and Rosa, 2006) and reaches adult levels in motor areas (Homman-Ludiye and Bourne, 2014). At 3 months postnatal (PM3), maturation is complete in the motor cortex and advanced in the visual cortex, with NNF now apparent in prefrontal areas (Burman et al., 2007). At 9 and 12 months (PM9 and PM12), NNF expression intensifies in prefrontal areas, including the DLPFC. By 18 months (PM18), all cortical areas reach peak maturation.

This study focuses on six PFC areas (Fig. 1B). Areas 8aD and 8aV, part of the frontal eye fields, control saccadic eye movement (Bruce et al., 1985). Their maturation has been explored using NNF (Burman et al., 2007). Compared to areas 9 and 46, their posterior position helps evaluate the hypothesis that cortical areas mature in a posterior-to-anterior manner (Gogtay et al., 2004). Areas 47 (lateral prefrontal cortex; granular) and 11 (orbitofrontal cortex; granular) play roles in cognitive flexibility and decision-making (Burke et al., 2014; Tremblay and Schultz, 1999; Rudebeck and Murray, 2014; Levy and Wagner, 2011).

The lissencephalic surface of the marmoset brain allows for isotropic growth and comparison of cortical demarcation at various stages. Cytoarchitectural boundaries were demarcated based on literature (Burman et al., 2006, 2007; Burman and Rosa, 2009), using Hoechst staining (Fig. 1C). For example, L4 in 8aV is thicker than in 8aD, delineated by the relative expansion of L3 and L4 and a sharp interface with L5. Area 46 has a well-defined but thinner L4. Area 47L, lateral to area 46, has a thinner L4 and thicker infragranular layers. Area 9 has a thin L4 and homogeneous layers. Area 11, with the thinnest L4, occupies the anterior orbital sulcus.

### Synchronous postnatal decrease of SST^+^ INs in the prefrontal areas

**Fig. 2.**
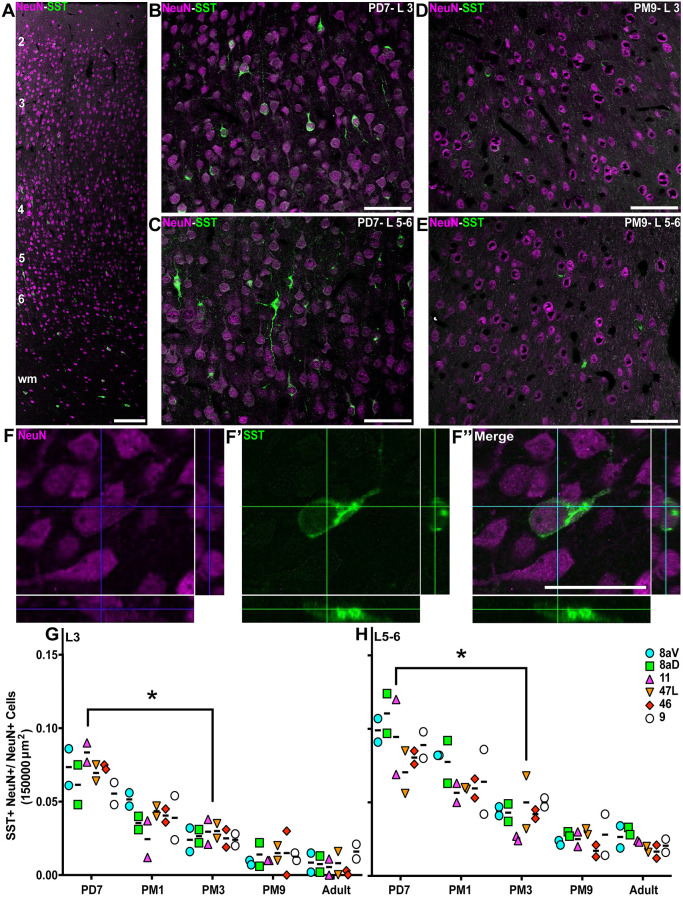
**Progressive reduction of the proportion of SST^+^ INs in the marmoset PFC from birth to adolescence.** (A) SST^+^ INs (green) and NeuN^+^ neurons (magenta) are present across all cortical layers of the marmoset DLPFC (area 46) at PD7. wm, white matter. Image stitching was performed to generate the micrograph of full cortical depth. (B-E) Distribution of SST^+^ NeuN^+^ INs in area 46 L3 and L5-6 at PD7 (B,C) and PM9 (D,E). (F-F″) Cutlines through a 3D stack acquired with a laser scanning confocal microscope confirm that NeuN nuclear labeling and cytoplasmic SST labeling correspond to a unique cell in area 46 L3 at PD7. (G,H) The ratio of SST^+^ NeuN^+^ INs over the total number of NeuN^+^ neurons per 150,000 µm^2^ was calculated at each developmental stage in all six PFC areas of interest and plotted over time. The proportion of SST^+^ INs decreases progressively and at a comparable rate in all areas in the supragranular L3 (G) and L5-6 (H). Statistical analysis was performed using a nonparametric Kruskal–Wallis test followed by Dunn's multiple comparisons. The data are presented as median. *P<0.05 (*n*=2). Scale bars: (A) 200 µm; (B-E) 50 µm; (F″) 10 µm.

SST is widely expressed across the brain, particularly in a subset of cortical INs. SST^+^ INs are one of the two subgroups of MGE-derived cortical INs, with the PV^+^ subtype comprising most cortical INs across mammals, including primates (Krienen et al., 2020). While the timing of incorporation of SST^+^ cells in neuronal networks in primates has not yet been elucidated, rodent studies suggest an earlier integration of SST^+^ INs into functional networks, compared to PV^+^ cells (Su et al., 2020; Tuncdemir et al., 2016). We observed SST^+^ NeuN (RBFOX3)^+^ INs in the marmoset PFC as early as the first postnatal week in supragranular and infragranular layers, and the white matter (Fig. 2A-C), which had declined in all areas and layers by adolescence (Fig. 2D,E). To substantiate our observation, we counted the density of SST^+^ in L3 and in L5 and L6 (L5-6) for each time point. We normalized our values to the total number of neurons in each counting frame to account for cortical expansion due to the addition of glial cells and synapses during postnatal development. After confirming that the proteins were co-expressed within the exact INs, we calculated the SST^+^ NeuN^+^/total NeuN^+^ neuron ratio in the field of view (Fig. 2F-F″).

First, the relative distribution of SST across all PFC areas at each time point was insignificant. At PD7, SST^+^ INs accounted for 0.074±0.02 of L3 neurons (Fig. 2G). This value was similar across all areas of the prefrontal cortex examined. The ratio rapidly decreased over development, dropping to 0.027±0.01 at PM3 (*P*≤0.01, Kruskal–Wallis test). The ratio of SST^+^ INs did not significantly vary during adolescence and adulthood, and the changes were homogeneous across all PFC areas of interest. Analysis of L5-6 yielded a comparable profile (Fig. 2H), with the exception that the ratio of SST^+^ INs over the total neuronal population was higher in the first postnatal week (0.09±0.03) and decreased to 0.05±0.02 (*P*≤0.01, Kruskal–Wallis test) at PM3.

To confirm that the developmental reduction in SST^+^ INs did not result from apoptotic cell death at PM3, and was more likely a downregulation of the peptide, activated caspase 3 (aCasp3) labeling and nuclear Hoechst stain (to identify pyknotic cells) were used (Fig. S1A). The results revealed a significant decrease in the population of aCasp3^+^ Hoechst^+^ cells in both L3 and L5-6 of areas 46 and 47L between PD7 and PM3 (Fig. S1B,C). However, the fraction of aCasp3^+^ SST^+^ cells remained consistent between these stages in the supra- and infragranular layers of areas 46 and 47L (Fig. S1D,E), suggesting that apoptosis may not account for the developmental reduction of the majority of SST^+^ INs between these stages.

Using *in situ* hybridization data available online in the Marmoset Gene Atlas (Kita et al., 2021; Shimogori et al., 2018), we illustrated *SST* mRNA in area 8aV and in V1 at PD0 (birth) and 1 year of age (Fig. S1F). In harmony with the SST^+^ NeuN^+^ cell quantification data (Fig. 2), *SST* mRNA appeared to reduce between birth and adulthood in the PFC and V1. To test whether the fraction of SST^+^ NeuN+ cells decreases in V1, we quantified SST^+^ NeuN^+^ cell numbers in the marmoset V1 across postnatal development (Fig. S1G-L). In general, there seemed to be a reduction trend in the cell numbers in V1; however, in contrast with the PFC (Fig. 2), it was not identified to be significant between PD7 and PM3 in both upper and inner layers (Fig. S1K,L). These data support the spatiotemporal variability of the SST expression profile between the PFC and V1.

To address whether the decline in the population of SST^+^ INs is linked to a developmental event, we used medial pulvinar (PM) thalamic lesioned marmoset models developed in the Bourne laboratory (Mundinano et al., 2016), in which the PM was ablated during the neonatal stage (PD14-18) (Mundinano et al., 2016). PM is a primate-specific, higher-order thalamic structure that establishes a dense, reciprocal connectivity network with numerous associative cortical areas, especially the PFC (Homman-Ludiye and Bourne, 2019; Homman-Ludiye et al., 2020). The exact function of this connectivity has not been well studied, but it is presumed to play a modulatory role and be involved in directing selective attention. We previously observed that neonatal manipulation of this thalamic region is linked to a decrease in the density of PV^+^ INs in adulthood and cognitive dysfunctions related to the disruption of the PFC-PM circuit (J.T.S., N.S.H.F. and J.A.B., unpublished). Here, we investigated whether the fraction of SST^+^ INs is altered in adult animals (>2.5 years) who received a lesion of PM neonatally (referred to hereafter as lesioned animals). Quantification of SST^+^ NeuN^+^ cells in the PFC of lesioned animals (areas 47L and 46) did not support a change in the number of SST^+^ NeuN^+^ cells in the PM-lesioned group compared to the control (Fig. S1M-R), ruling out a role of the PM-PFC connectivity network in the maturation of SST^+^ cells. Using this experiment, we provided mechanistic insights into our previous observations relating to SST^+^ cell modifications during postnatal development.

These findings suggest that the maturation of SST^+^ INs is synchronized across the PFC, unlike the sensory cortex. Considering this result, we investigated the developmental regulation of PV expression, the other primary class of cortical MGE-derived INs.

### A proportional increase in the fraction of PV^+^ INs in the prefrontal areas during preadolescence

**Fig. 3.**
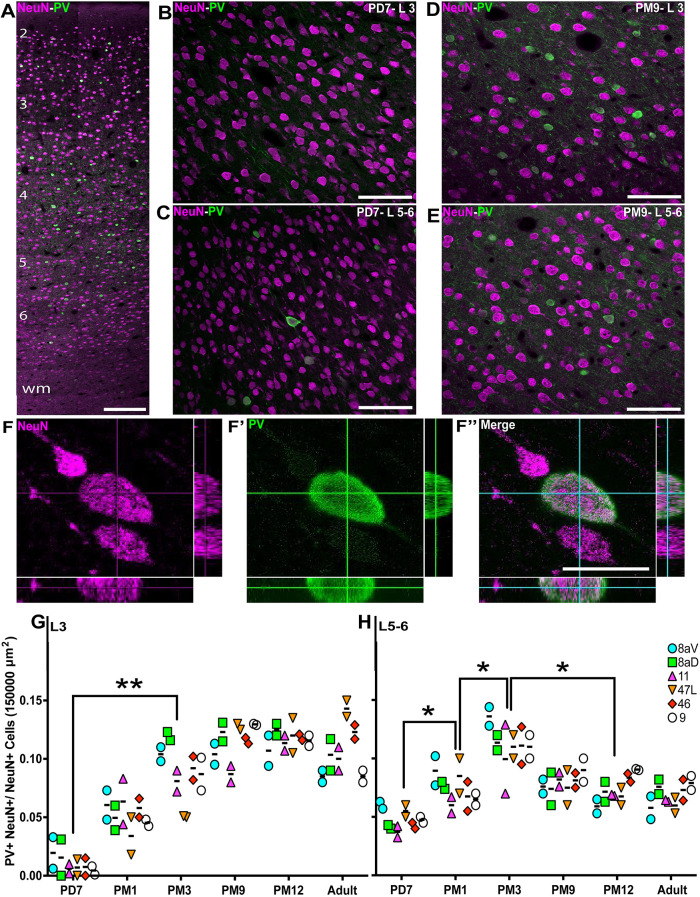
**Progressive increase of PV^+^ INs in the marmoset PFC from birth to adolescence.** (A) PV^+^ INs (green) and NeuN^+^ neurons (magenta) are present across all cortical layers of the marmoset DLPFC (area 46) at PD7. wm, white matter. (B-E) Distribution of PV^+^ NeuN^+^ INs in area 46 L3 and L5-6 at PD7 (B,C) and PM9 (D,E). (F-F″) Cutlines through a 3D stack acquired with a laser scanning confocal microscope confirm that NeuN nuclear labeling and cytoplasmic PV labeling correspond to a unique cell in area 46 L3 at PD7. (G,H) The ratio of PV^+^ NeuN^+^ INs over the total number of NeuN^+^ neurons per 150,000 µm^2^ was calculated at each developmental stage in all six PFC areas of interest and plotted over time. The proportion of PV^+^ INs increases progressively and at a comparable rate in all areas in the supragranular L3 (G). In the L5-6 (H), the proportion of PV^+^ NeuN^+^ initially increased, like L3, followed by a reduction at the start of adolescence. Statistical analysis was performed using a nonparametric Kruskal–Wallis test followed by Dunn's multiple comparisons. The data are presented as median. *P<0.05, **P<0.01 (*n*=2). Scale bars: (A) 200 µm; (B-E) 50 µm; (F″) 10 µm.

PV^+^ INs were observed primarily in L3-5 (e.g. Fig. 3A, area 46 at PM9). At PD7, PV^+^ INs were largely absent from L3 (Fig. 3B) and more prominent in L5-6 (Fig. 3C). By PM9, the density of PV^+^ cell profiles had noticeably increased (Fig. 3D,E), and neuropil labeling was perpendicular to the surface.

We confirmed that PV^+^ INs expressed the pan-neuronal transcription factor NeuN (Fig. 3F-F″) and proceeded to quantify the ratio of PV^+^ INs. The results in L3 confirmed our initial observations, revealing very few PV^+^ cells at PD7 across all PFC areas observed (0.003±0.0007; Fig. 3G). The proportion increased rapidly over the next 3 months (0.094±0.06; *P*≤0.005, Kruskal–Wallis test), stabilizing to adult levels around 9 months old.

For L5-6, the average proportion of PV^+^ INs was greater at PD7 (Fig. 3H; 0.045±0.02). It increased over the first 3 months of life, peaking at PM3 (0.12±0.03; *P*≤0.02, Kruskal–Wallis test), followed by a reduction during adolescence (0.07±0.03; *P*≤0.02, Kruskal–Wallis test), then remaining constant into adulthood (0.07±0.02). While deep cortical layers exhibited a distinct profile from superficial L3, all PFC areas revealed a similar profile in distribution, suggesting a common regulatory mechanism. These analyses also revealed that the fraction of PV^+^ INs is more significant than that of SST^+^ across all developmental stages observed.

**Fig. 4.**
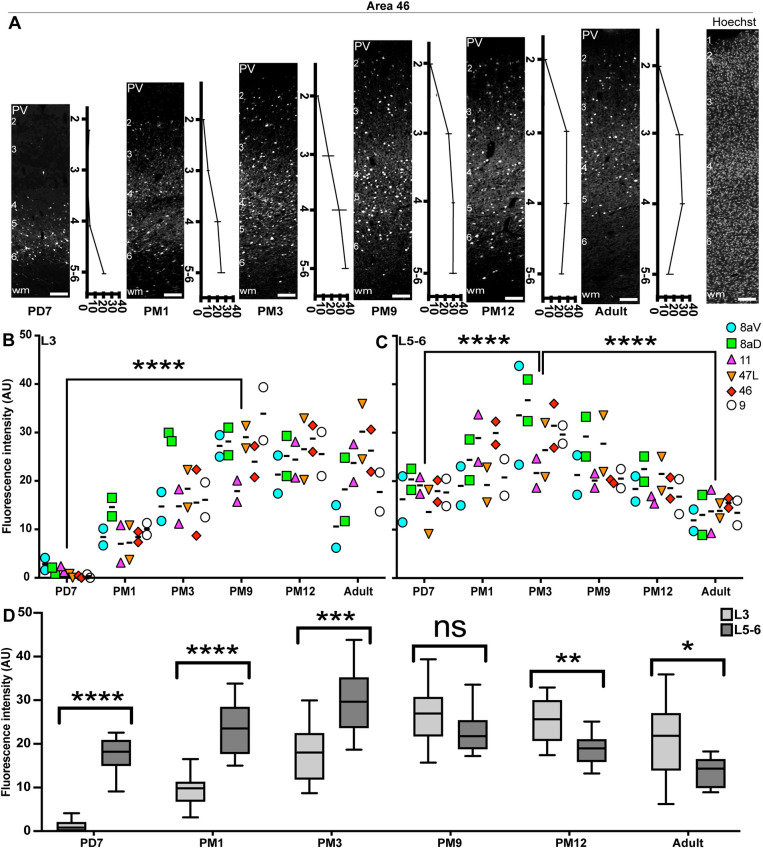
**Cellular and neuropil expression of PV consistently increases from birth to adolescence across the marmoset PFC.** (A) Laminar distribution of PV immunofluorescence (normalized to L1) across different ages in area 46. Hoechst labeling was used to demarcate the individual cortical layers. wm, white matter. Image stitching was carried out to generate cortical image strips. (B,C) Quantification of PV signal intensity revealed a consistent increase in PFC L3 and L5-6 from birth to adolescence. While levels remained stable in L3 during adulthood, they decreased in L5-6 from PM9 to adult. The decrease in PV fluorescence intensity from PM3 until adulthood was statistically significant. Data are presented as median. (D) Comparison of the PV immunofluorescence signal intensity in L3 and L5-6 in areas 8aD, 8aV, 47, 11, 9 and 46. The box plot visualizes the distribution of PV immunofluorescence intensity across prefrontal areas at different developmental stages and layers. The central horizontal line represents the median value; the box itself spans from the 25th percentile (bottom edge) to the 75th percentile (top edge), containing the middle 50% of all measurements; and the whiskers extend to the minimum and maximum recorded values, showing the complete data range for each age/layer. Statistical analysis was performed using a nonparametric Mann–Whitney test. Data are presented as median±interquartile ranges. *P<0.05, **P<0.01, ***P<0.001, ****P<0.0001 (*n*=2). AU, arbitrary units; ns, non-significant. Scale bars: 20 µm.

Establishing a functioning network of PV^+^ INs is not limited to the number of INs, which does not tend to vary between healthy and diseased brains in the context of SCZ or ASDs (Filice et al., 2020; Lewis et al., 2012), but also the level of PV expression, which can make these INs vulnerable to stressors. To assess expression levels, we calculated the intensity of the PV signal across all cortical layers. To account for background noise, values were normalized to the intensity of the signal in L1, devoid of PV expression. For example, the signal in area 46 was exclusively located in L5-6 at PD7 (Fig. 4A), progressively expanding in L4, L3 and L2 over the first 3 months of life. The signal intensity, comprising cell body and neuropil immunostaining, was greatest during adolescence (PM9-PM12), remaining weaker in L2 than in all the other layers. In the adult, the intensity remained strong in L3 and L4 but dropped in L5-6, corresponding with the reduction of cell body count described above.

We applied this method to all areas of interest in the PFC and plotted the average value for L3 (Fig. 4B, Fig. S2) and L5-6 (Fig. 4C, Fig. S2). The results followed the same profile as the cell counts, suggesting a correlation between cell counts and PV intensity across the developmental stages examined.

We then compared the intensity of the PV signal in L3 and L5-6 for each developmental stage (Fig. 4D), confirming that the fluorescence in L3 was consistently lower until the beginning of adolescence (PM9), at which point an inflection occurred and L3 became predominant. This suggests that adolescence is a turning point in establishing adult PV function.

### Consolidation of PV^+^ IN connectivity in the PFC occurs during adolescence

**Fig. 5.**
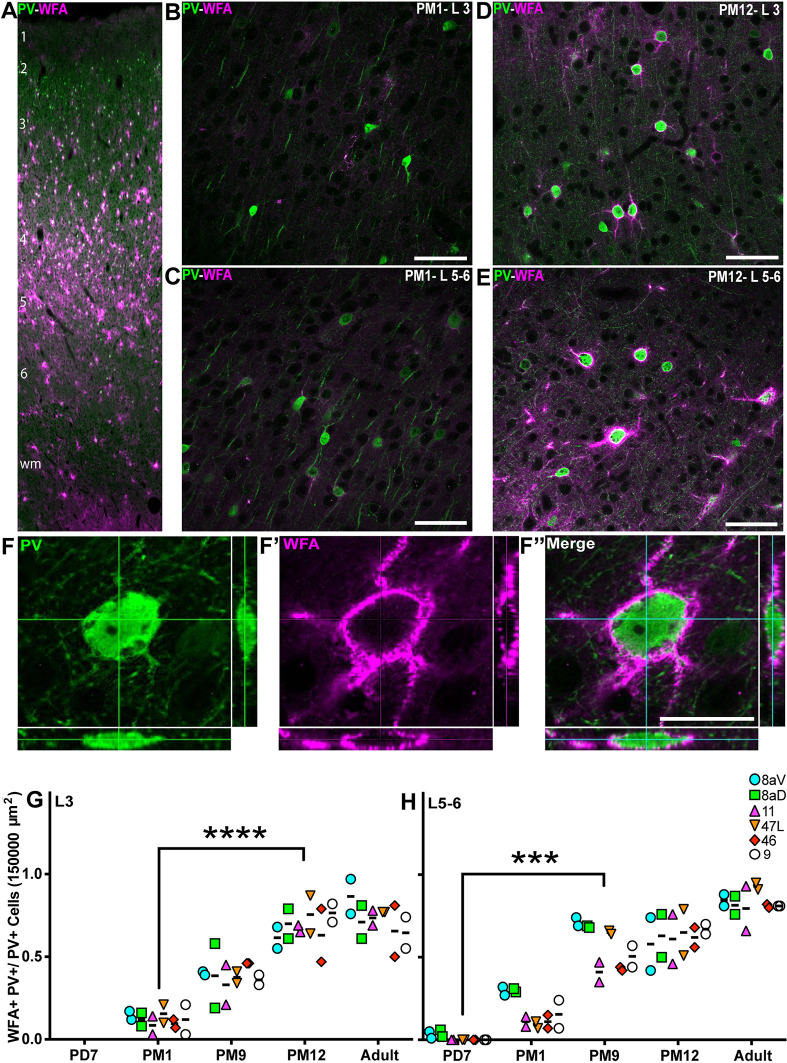
**Progressive accumulation of WFA^+^ perineuronal nets on PV^+^ dendritic tree from birth to adult across the marmoset PFC.** (A) PV^+^ INs (green) and WFA^+^ membrane (magenta) are present across all cortical layers the marmoset DLPFC (area 46) at PD7. Image stitching was performed to generate the micrograph of full cortical depth. wm, white matter. (B-E) Distribution of WFA^+^ PV^+^ INs in area 46 L3 and L5-6 at PM1 (B,C) and PM12 (D,E). (F-F″) Cutlines through a 3D stack acquired with a laser scanning confocal microscope confirm that PV cytoplasmic labeling and membrane-bound WFA labeling correspond to a unique cell in area 46 L3 at PM12. (G,H) The ratio of WFA^+^ PV^+^ INs over the total number of PV^+^ neurons per 150,000 µm^2^ was calculated at each developmental stage in all six PFC areas of interest and plotted over time. The proportion of WFA^+^ INs increases progressively and at a comparable rate in all areas in the supragranular L3 (G) and infragranular L5-6 (H). Statistical analysis was performed using a nonparametric Kruskal–Wallis test followed by Dunn's multiple comparisons. The data are presented as median. ***P<0.001, ****P<0.0001 (*n*=2). Scale bars: (A) 200 µm; (B-E) 50 µm; (F″) 10 µm.

The ultimate step of cortical maturation corresponds to the consolidation of the synaptic connections that persist beyond the phase of synaptic pruning (Kirchner et al., 2025). Consolidation consists of two significant events: axonal myelination (Simons and Trotter, 2007) and the deposition of chondroitin sulfate proteoglycans (CSPG), one of the main components of PNNs (Browne et al., 2022). The CSPG scaffold encapsulates the pre- and postsynaptic elements, creating a mesh-like structure that stabilizes the synapse, which occurs late in development. It correlates with establishing mature synapses following a ‘critical period’. PNNs are heterogeneously enriched across the neocortex, their distribution profile enabling the parcellation of discrete cortical areas (Homman-Ludiye et al., 2010). To assess the level of CSPG scaffold, PV^+^ synapses in the marmoset PFC, we used *Wisteria floribunda* agglutinin (WFA) labeling to reveal the accumulation of CSPG in PNNs throughout postnatal development and into adulthood. For area 46, WFA cell-profile labeling was observed predominantly in L3-5 and the white matter in adulthood (Fig. 5A). At PM1, faint WFA labeling could be observed in L3 but did not correlate with PV^+^ INs (Fig. 5B). However, in L5-6, WFA colocalized with PV labeling (Fig. 5C). At 12 months old, most PV^+^ INs were outlined by WFA labeling, including their proximal neurites in L3 (Fig. 5D) and L5-6 (Fig. 5E). High-magnification confocal images of PV/WFA double-positive INs illustrate how PNNs thickly envelop the cell body and the extensive proximal neurites (Fig. 5F-F″).

We further quantified the fraction of PV^+^ INs cloaked with WFA to support these observations. In L3, the WFA^+^ PV^+^ cells were observed at PM1. The proportion of double-positive PV^+^ INs steadily increased over development, reaching a plateau by PM12, when over 80% of PV^+^ INs exhibited PNNs, consistent across all PFC areas of interest (Fig. 5G). The time course of PNN accumulation on PV^+^ INs in L5-6 preceded that of L3 (Fig. 5H) as the first WFA^+^ PV^+^ double-positive INs were observed as early as PD7 but otherwise followed a comparable time course, peaking between PM12 and adulthood. Interestingly, areas 8aV and 8aD revealed an earlier peak of WFA^+^ PV^+^ INs in L5-6 at PM9 compared to other PFC areas.

Additionally, the precise changes in fluorescence intensity of WFA around the cell membrane of PV^+^ cells were investigated in three prefrontal areas: 11, 47L and 46 (Fig. S3A,B). The data analysis revealed a progressive increase in WFA fluorescence intensity from PM1, reaching an adult-like level in PM12 (21.6±16.6 versus 380.8±40; *P*=0.01, Kruskal–Wallis test) (Fig. S3A). Likewise, in L5-6, the fluorescence intensity of WFA significantly increased from PD7 to PM12 (2.6±4.3 versus 524±155; *P*=0.01). A further 64% increase was detected from PM12 until adulthood. However, it was not identified to be statistically significant (524±155 versus 861.5±222; *P*>0.9) (Fig. S3B). These findings are consistent with cell counting data indicating that the accumulation of PNNs around PV^+^ cells was a protracted process until mid-adolescence.

To investigate the modifications of a fraction of PV^+^ WFA^+^ cells among WFA^+^ cells, the ratio of PV^+^ WFA^+^/WFA^+^ cells was quantified in the PFC areas 11, 47L and 46 across postnatal development (Fig. S3C,D). We detected a gradual increase in the population of PV^+^ WFA^+^/total WFA^+^ cells from PM1 until PM12 (0.59±0.15 versus 0.88±0.1; *P*=0.004, Kruskal–Wallis test) in L3 of the studied areas (Fig. S3C), which correlated with the postnatal increase in the population of PV^+^ INs (Fig. 3G) and PV^+^ WFA^+^ cells (Fig. 5G) observed previously. A distinct pattern emerged in L5-6: the fraction of PV^+^ WFA^+^/WFA^+^ cells remained stable from PM1 to PM9 (*P*>0.05) (Fig. S3D), supporting an earlier maturation of PV^+^ INs in infragranular layers (Fig. 3H), interestingly, this population declined from PM9 to adulthood (0.88±0.13 versus 0.61±0.1; *P*=0.006) (Fig. S3D), which possibly reflects the delayed maturation of other neuronal subtypes through the PNN deposition mechanism.

### Upregulation of KCC2 in the cell membrane of PV^+^ INs in infancy

The upregulation of PV in young INs is presumed to occur through activity-dependent pathways (Chung et al., 2017) to increase Ca^+2^ buffering capability, leading to burst firing (Hijazi et al., 2023). For this reason, PV is a reliable indicator of IN maturation. However, PV is not unique, and other cellular aspects can predict functional maturation. One such marker is the potassium chloride co-transporter KCC2. For example, as PV^+^ INs mature, there is a significant upregulation of KCC2. This increase in KCC2 expression lowers the intracellular chloride concentration, allowing GABAergic inputs to become hyperpolarizing and thus inhibitory (Herrmann et al., 2022; Ben-Ari, 2002).

**Fig. 6.**
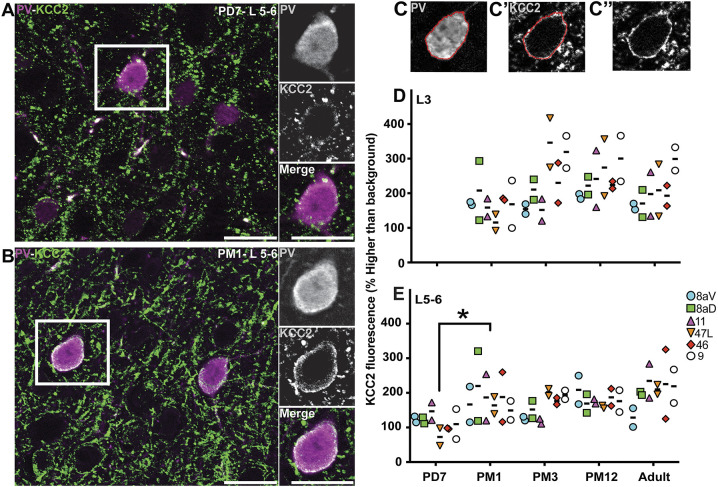
**Expression of KCC2 in PV^+^ cells and prospective PV^+^ INs from birth to adulthood in the marmoset PFC.** (A,B) PV (magenta) and KCC2 (green) labeling in L5-6 of area 46 at PD7 (A) and PM1 (B). (C-C″) Representative example of the quantification of KCC2 fluorescence intensity on the membrane of a PV^+^ interneuron. First, the outline of the PV^+^ cytoplasm is traced (C), and this outline pasted on the channel corresponding to KCC2 labeling (C′) to measure the fluorescent signal intensity. C″ shows the same image without the outline. (D,E) The intensity of the KCC2 fluorescent signal intensity on PV^+^ INs of the marmoset PFC L3 (D) and L5-6 (E) was plotted across development. Statistical analysis was performed using the nonparametric Kruskal–Wallis test, followed by Dunn's multiple comparisons. Data are presented as median. *P<0.05 (*n*=2). Scale bars: 20 µm.

To determine the extent to which KCC2 informs the maturation of INs during the postnatal development of the marmoset PFC, we analyzed the expression of KCC2 on PV^+^ INs. At PD7, we observed the presence of KCC2 puncta on the putative cell membrane of PV^+^ cell profiles in L5-6 (Fig. 6A), which was more uniform and ubiquitous than the staining observed at PM1 (Fig. 6B). To confirm this observation, we quantified the intensity of the KCC2 signal along the outline of PV^+^ cell bodies (Fig. 6C-C″), as previously described (Akhter et al., 2019).

In L3, the amount of KCC2 signal around PV^+^ INs did not vary across development or areas (Fig. 6D). In L5-6, the amount of KCC2 signal on PV^+^ INs increased twofold between PD7 and PM1 (Fig. 6E), then remained constant into adulthood.

The hypothesis suggesting that the upregulation of PV is linked to neuronal activity has previously been investigated in the context of disease conditions, such as epilepsy. However, no direct evidence suggests that neuronal activity derives from early developmental PV upregulation. Here, the observed steady KCC2 signal around PV^+^ INs through development in L3 suggests that KCC2 upregulation might precede that of PV. To test this hypothesis, we identified a combination of proteins to label a presumptive subgroup of PV^+^ INs, namely Sox6 and TAC1. The transcription factor Sox6 is expressed in all INs emerging from the MGE (Batista-Brito et al., 2009). TAC1 is a neuropeptide notably involved in increasing cytosolic Ca^+2^ ions (Nässel et al., 2019) and is expressed in various neuronal fractions, including PV^+^ INs (Pfeffer et al., 2013). We randomly selected area 8aD, tested the hypothesis in L3 at PD7 (when there are few PV^+^ cells in L3; see Fig. 3), and identified distinctive KCC2^+^ puncta outlining the surface of putative PV^+^ INs (Fig. S4). These data suggest that KCC2 expression precedes that of PV.

### Peak expression of a fast-spiking phenotype in PV^+^ INs during postnatal development

**Fig. 7.**
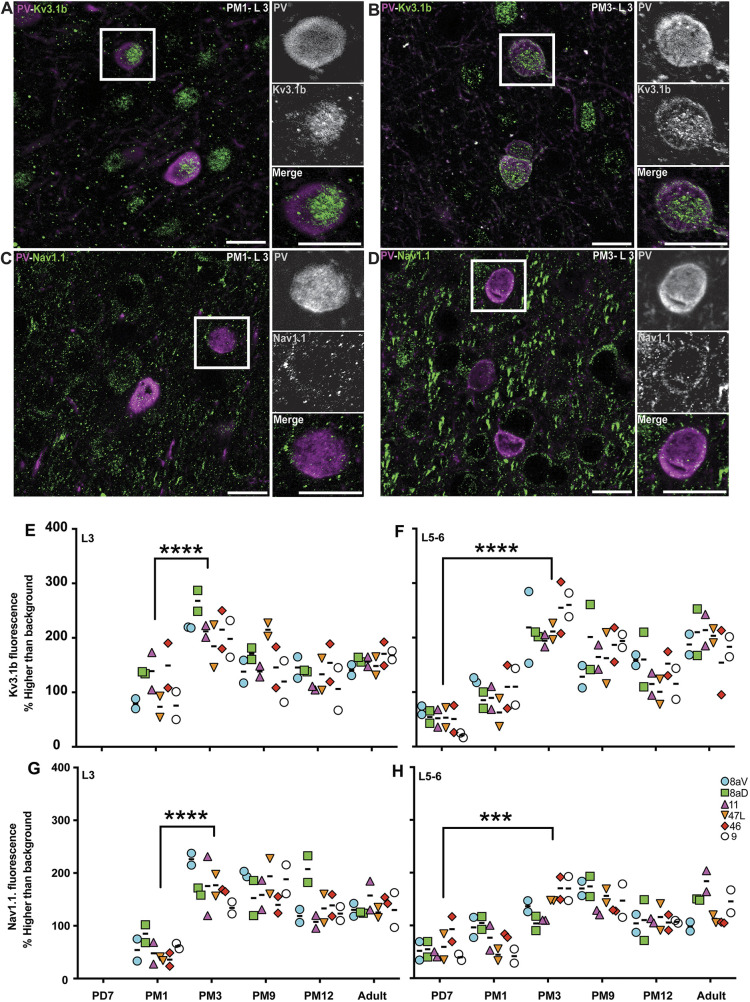
**Expression of Kv3.1b and Nav1.1 ion channels progressively increases on the membrane of PV^+^ neurons across the developing marmoset PFC**. (A,B) Kv3.1b (green) and PV (magenta) expression in INs in L3 of area 46 at PM1 (A) and PM3 (B). (C,D) Nav1.1 (magenta) and PV (green) expression in INs in area 46 L3 at PM1 (C) and PM3 (D). (E,F) Intensity of Kv3.1b fluorescent signal in PV^+^ INs in L3 (E) and L5-6 (F) of the marmoset PFC plotted over time reveals an increase during the first 3 postnatal months. (G,H) Intensity of Nav1.1 fluorescent signal in PV^+^ INs in L3 (G) and L5-6 (H) of the marmoset PFC plotted over time reveals an increase during the first 3 postnatal months. Statistical analysis was performed using the nonparametric Kruskal–Wallis test, followed by Dunn's multiple comparisons. Data are presented as median ranges. ***P<0.001, ****P<0.0001 (*n*=2). Scale bars: 20 µm.

Mature cortical PV^+^ INs generate fast-spiking action potentials. This characteristic is crucial for normal function of the adult neocortex. The potassium channel Kv3.1b and the sodium channel Nav1.1 are essential for generating fast-spiking activity in PV^+^ INs (Gu et al., 2018). To estimate the postnatal stage at which PV^+^ INs have the capacity for fast-spiking action potentials, we used the same analytical approach as for KCC2 to analyze the distribution of Kv3.1b and Nav1.1 on PV^+^ INs in the maturing PFC. Kv3.1b was expressed at the surface of PV^+^ INs in L3 as early as 1 month of age (Fig. 7A) and persisted at 3 months (Fig. 7B). We also observed Nav1.1 expression around PV^+^ INs at PM1 (Fig. 7C) and PM3 (Fig. 7D).

Quantitative analysis across all areas of interest revealed that Kv3.1b was not detected on PV^+^ INs at PD7 in L3, but the quanta of Kv3.1b on PV^+^ INs doubled between PM1 and PM3 (Fig. 7E). Kv3.1b signal appeared to decrease during adolescence. In L5-6, Kv3.1b signal was present around PV^+^ INs from PD7 onwards, increasing steadily over the first 3 postnatal months and remaining constant throughout adolescence and adulthood (Fig. 7F).

The profile of Nav1.1 expression was comparable to that of Kv3.1b, with no signal observed at PD7 in L3 and a doubling of the intensity of the signal between PM1 and PM3 (Fig. 7G). The expression remained constant from PM3 to adulthood. In L5-6, Nav1.1 was detected around PV^+^ INs from PD7, increasing over the first 3 postnatal months (Fig. 7H), although to a lesser extent than Kv3.1b.

**Fig. 8.**
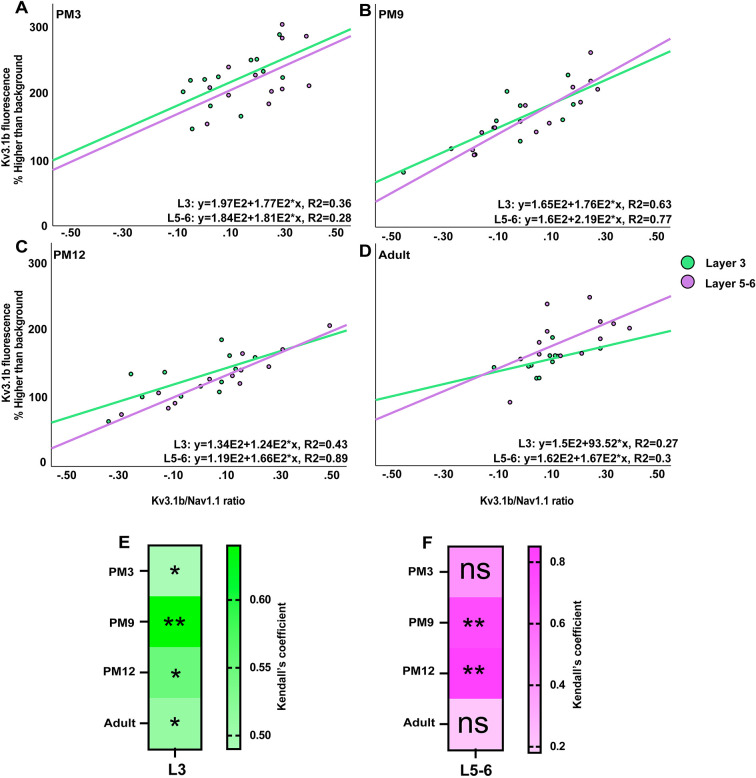
**Highest correlations between factors essential for fast-spiking properties of PV^+^ INs during adolescence.** (A-D) Kendall's correlation between the fluorescence intensity of Kv3.1b and ratio of Kv3.1b to Nav1.1 in L3 (green) and L5-6 (magenta) of PFC at PM3 (A), PM9 (B), PM12 (C) and adulthood (D), at which the highest correlation was observed (see Fig. 7). (E,F) Heat maps highlight PM9 as the age during which Kendall's correlation coefficient was the highest consistently in L3 (E) and L5-6 (F), suggesting that maturation of the fast-spiking phenotype of PV^+^ INs occurs during adolescence. *P<0.05, **P<0.01 (*n*=2). ns, not significant.

Analysis of transcriptomics and electrophysiological data has previously demonstrated a strong positive correlation between (1) high expression of Kv3.1b and (2) a balanced index calculated by the Kv3.1b/Nav1.1 ratio, which determines the fast-spiking phenotype of PV^+^ INs (Gu et al., 2018). To predict the age during postnatal development at which the fast-spiking phenotype of PV^+^ cells may be the highest, we applied the Kendall correlation test between the Kv3.1b expression level and the Kv3.1/Nav1.1 ratio in L3 and L5-6 between PM3 and adulthood (Fig. 8A-D). In L3, the Kendall test revealed the highest correlation coefficient at PM9 (coefficient value=0.63) (Fig. 8B,E). The correlation coefficient decreased by a fraction of 0.43 in adulthood compared to PM9 (Fig. 8B,D,E). In L5-6, significant correlation coefficients were also detected during adolescence stages, PM9 and PM12 (coefficient values of 0.77 and 0.89, respectively) (Fig. 8B,C,F), and a decrease of 66% was evident in adulthood compared with PM12 (Fig. 8C,D,F).

## DISCUSSION

This study examined the postnatal maturation trajectory of MGE-derived INs, including PV^+^ and SST^+^ subtypes, in the marmoset PFC. The intricate development of the PFC involves diverse cellular and molecular mechanisms, hypothesized to undergo a temporal delay relative to sensorimotor cortex maturation. Failures in PFC molecular and cellular processes are linked to several neurodevelopmental disorders (Yanagi et al., 2014; Kaar et al., 2024; Yamakawa, 2016; Woodbury-Smith et al., 2022; Hyde et al., 2011; Sullivan et al., 2015; Lam et al., 2023). INs are crucial for maintaining neural activity balance, regulating the timing and synchronization of neural signals, and fine-tuning neural circuitry (Tremblay et al., 2016). We observed a similar maturation profile across PFC areas (8aD, 8aV, 9, 11, 46 and 47). However, SST^+^ INs peaked by PD7, whereas PV neurons peaked by adolescence (PM9). Considering the protracted development of PV neurons and their role in higher cognitive functions (Fung et al., 2010; Hashimoto et al., 2008; Mellios et al., 2009; Filice et al., 2020; Ruden et al., 2021; Scheuer et al., 2021; Shi and Bergson, 2020; Lauber et al., 2016; Juarez and Martínez Cerdeño, 2022; Hashemi et al., 2017; Ariza et al., 2018), we elucidated the spatiotemporal upregulation of ion channels KCC2, Kv3.1b and Nav1.1, which are intricately associated with PV^+^ IN functions.

### Unraveling the discrepancy in the maturation of prefrontal cortex SST^+^ INs: primate versus rodent

The developmental profile of SST^+^ INs in the PFC is still debated and may be species specific. We found that in the marmoset PFC the fraction of SST^+^ INs is highest in the first postnatal week, decreases until adolescence, and then stabilizes. This aligns with human data revealing a gradual postnatal decrease in *SST* mRNA in the PFC (Fung et al., 2010), whereas mouse medial PFC numbers remain stable over the first 40 days (Bitzenhofer et al., 2020). These results suggest a divergence between primate and rodent SST^+^ INs in the PFC.

Our observations, coupled with the evolutionary enlargement of the PFC and incorporation of granule cell layer regions, suggest a unique postnatal IN developmental progression in the primate granular PFC compared to rodents and sensory cortex. These findings have significant implications for selecting appropriate animal models for studying neurodevelopmental disorders such as SCZ, ASDs and ADHD.

### Accelerated maturation of SST^+^ INs compared to PV^+^ INs in the monkey PFC

The rapid decline in SST^+^ INs suggests that SST influences early neuronal circuit processes. SST is a multifunctional hormone linked to migration (Jung et al., 2006), regulation of proliferation (Guillermet-Guibert et al., 2007), apoptosis (Hou and Yu, 2013), differentiation (Guillermet-Guibert et al., 2007), synaptogenesis and axon pathfinding (Ferriero et al., 1994) in maturing neuronal circuits. These functions are crucial during early postnatal development. In the mouse somatosensory cortex, SST^+^ INs strongly innervate PV^+^ INs at P6 in L5-6, acting as placeholders for establishing thalamocortical connectivity in PV^+^ INs (Tuncdemir et al., 2016; Marques-Smith et al., 2016). This supports the earlier maturation of SST^+^ INs compared to PV^+^ INs by mediating thalamocortical inputs onto PV cells (Dwivedi et al., 2024). *In situ* hybridization in marmosets shows widespread *SST* mRNA across PFC laminae at birth (PD0), with no *PV* mRNA detected (Shimogori et al., 2018; Kita et al., 2021). A study in human DLPFC demonstrated significant *PV* (*PVALB*) mRNA upregulation between birth and juvenility, compared to a decreasing trend of *SST* mRNA (Fung et al., 2010). This suggests that SST^+^ INs appear before PV^+^ INs in primates, including humans.

One likely reason for this observation may rely on the specialized functional contribution of IN subtypes in prefrontal circuits. It has been demonstrated that PV^+^ and SST^+^ subtypes exhibit distinct tendencies to connect with long-range excitatory projections from various cortical areas (Naskar et al., 2021). Naskar and colleagues reported that PV^+^ cells in rodent primary somatosensory area (S1) receive predominantly long-range cortical inputs from other somatosensory areas to enable feed-forward inhibition of pyramidal neurons. In contrast, long-range cortical connections project weakly to the SST^+^ subtype. Although these findings need to be examined in the PFC, the evidence suggests that prefrontal areas establish a dense network of cortical interconnectivity (Majka et al., 2016) to perform complex computations and enable higher cognitive functions. Therefore, it is plausible that the number of PV^+^ cells increases postnatally to support the maturation and regulation of this complexity. Interestingly, although differences exist between primates and rodents, PV upregulation around adolescence is also seen in the medial PFC of rats (Petros et al., 2015). This supports the idea that PV is essential for refining prefrontal GABAergic function and connectivity, aiding the acquisition of the mature GABAergic phenotype necessary for adult PFC functions. In contrast, the SST^+^ IN subtype is thought to receive weak long-range cortical inputs (Naskar et al., 2021). Consequently, SST^+^ cells may decrease in number or downregulate SST expression after fulfilling their developmental roles, such as orchestrating cortical network desynchronization and facilitating PV^+^ IN maturation (Mòdol et al., 2024). However, the precise mechanism underlying the post-developmental reduction in SST^+^ cells remains unclear, whether due to cell loss or transcriptional downregulation of SST.

### The maturation of the PV^+^ IN network is synchronized across the cortices of the PFC

Our analysis of the PV^+^ IN subgroup, the largest IN fraction in the primate neocortex, revealed synchronized maturation across PFC areas. These findings align with the maturation of NNF^+^ pyramidal neurons (Burman et al., 2007), highlighting the role of the PV^+^ IN network in maturation. Notably, studies in the V1 of marmoset and macaque monkeys show adult-like PV^+^ INs levels as early as the neonatal stage (PD14 and PD22) (Mundinano et al., 2015; Condé et al., 1996). This contrasts with our findings, indicating protracted PV^+^ IN maturation in the PFC during early adolescence, suggesting spatiotemporal differences in PV expression in the non-human primate cortex.

The timing of PV^+^ IN maturation appears to be determined by their laminar identity, as the infragranular layer matures before the supragranular layer. For example, PV^+^ INs are present in L5-6 by the first postnatal week, but their presence in L3 is delayed. Similarly, PNN deposition on PV^+^ INs peaked by PM9 in L5-6 but only at PM12 in L3. This temporal profile matches that of NNF^+^ pyramidal neurons across the marmoset PFC and supragranular versus infragranular dichotomy (Burman et al., 2007). This might reflect the expression of PV on a fraction of pyramid-shaped neurons in L5 across mammals, including cats (Stichel et al., 1987), rats (van Brederode et al., 1991), marmosets (Spatz et al., 1994) and mice (Tanahira et al., 2009; Peng et al., 2021). While the excitatory phenotype of some pyramid-shaped PV^+^ cells has been confirmed in rodents, their description in primates is based on morphology in V1 (Johnson and Casagrande, 1995; Spatz et al., 1994). This subpopulation, which represents 10-20% of the PV^+^ population, was described in neonatal marmoset V1 and appeared to be transient, as it was absent in adult V1 (Spatz et al., 1994). Our observations revealed various morphologies, including some pyramid-like somata in the postnatal PFC, although their density was lower than in neonatal marmoset V1. Therefore, pyramid-shaped, potentially excitatory PV^+^ cells in the marmoset PFC likely form a negligible fraction of cells compared to typical PV^+^ INs.

Interestingly, not all PV^+^ INs exhibited PNN labeling in adulthood, suggesting that some may remain amenable to synaptic remodeling. Previous data indicate that adult PFC neurons can modify connectivity in response to environmental changes (Cook and Wellman, 2004; Perez-Cruz et al., 2007, 2009). In the mouse visual cortex, PV^+^ cells regulate plasticity in adulthood (Nahmani and Turrigiano, 2014), and PNN manipulation reinstates plasticity (Pizzorusso et al., 2002). These findings suggest that PNN modifications could regulate PV^+^ cell plasticity. Not all PV^+^ subtypes may express PNN components. In the human PFC, only basket cells among PV^+^ INs are surrounded by PNN, segregating them from other PV-expressing cells, such as chandelier cells (Ariza et al., 2018).

### Ion channel expression suggests that the fast-spiking phenotype of PV^+^ INs may be the highest in adolescent PFC

We observed that KCC2 expression preceded that of PV and was associated with putative PV^+^ INs during the first postnatal week, continuing into adulthood. This early KCC2 expression suggests that these INs already possess the capacity for hyperpolarizing GABA_A_ receptor currents. Increased PV protein expression during development has been linked to enhanced KCC2 function. Importantly, KCC2 dysfunction and Cl^−^ homeostasis disruption are implicated in neurodevelopmental disorders, including Down syndrome (Deidda et al., 2015), fragile X syndrome (He et al., 2014), Rett syndrome (Duarte et al., 2013; Tang et al., 2016) and SCZ (Arion and Lewis, 2011).

The hallmark of PV^+^ INs is their ability to generate fast-spiking action potentials, regulated by high Kv3.1b expression and a balance between Kv3.1b and Nav1.1 channels (Gu et al., 2018). Kv3.1b is selectively expressed in PV^+^ INs across rodents and primates (Rudy and McBain, 2001) and is required for rapid repolarization of PV IN membranes after sodium channel-mediated depolarization (Ben-Ari, 2002; Du et al., 1996; Kelly et al., 2019). Nav1.1 is also highly expressed in PV^+^ IN membranes and axon initial segments, contributing to depolarization and action potential propagation. Thus, Nav1.1 expression reflects neuronal activity within PV^+^ cells. Our findings show enhanced fast-spiking potential in PV^+^ INs across the supra- and infragranular layers of primate PFC during early adolescence.

The adolescent stage involves significant remodeling of PFC networks, followed by pathway consolidation (Pöpplau et al., 2024; Caballero et al., 2016; Larsen and Luna, 2018). The increased fast-spiking activity suggests PV^+^ IN involvement in adolescent PFC development. Supporting this, inhibition of PV^+^ cells during adolescence in rodents disrupts frontal cortex development and causes adult cognitive deficits (Canetta et al., 2022). These cells likely inhibit weak connections, facilitating immune-mediated synaptic pruning. During adolescence, the PFC also receives key excitatory inputs, including from the medial pulvinar and mediodorsal thalamus (Homman-Ludiye and Bourne, 2019). Disruption of mediodorsal input to mPFC during adolescence impairs its normal development (Benoit et al., 2022).

Rodent studies show enhanced PV^+^ fast-spiking firing and gamma oscillations during adolescence (PD25) (Bitzenhofer et al., 2020; Doischer et al., 2008; Yang et al., 2014). Similarly, robust gamma activity has been observed in the primate PFC during adolescence (Wang et al., 2022). Since gamma oscillations mediate cognitive functions such as working memory (Robbins, 2017; Sakurai and Gamo, 2019), adolescence likely represents a critical period of PFC maturation (Reynolds et al., 2019; Larsen and Luna, 2018). External and internal regulators of critical periods, such as BDNF, OTX2 and PNN, also promote PV^+^ cell maturation (Gibel-Russo et al., 2022; Itami et al., 2007). Thus, enhanced PV expression and fast-spiking properties during adolescence support their role in mediating this critical developmental window (Takesian and Hensch, 2013; Werker and Hensch, 2015).

### Statistical power versus temporal resolution

Our findings reinforce the discrepancy between rodents and primates in the functional/anatomical organization and cellular composition of the brain. This is especially true when investigating the PFC, where most areas and functions lack rodent equivalents. While research in developing nonhuman primates offers translatability that rodents cannot, it presents drawbacks, primarily cost and limited availability. For this study, we prioritized temporal resolution, using six developmental stages, over statistical power (*n*=2; 12 animals). Fortunately, minimal inter-areal variation allowed us to cluster regions and identify developmental trends. Like previous research (Burman et al., 2007), the small sample size limits speculation. Still, consistency across observations supports our conclusion that PFC areas mature uniformly, not sequentially. We also provide insights into PV^+^ IN physiology, including ion channel expression and PNN deposition.

## MATERIALS AND METHODS

### Animals

Twelve marmoset monkeys (*Callithrix jacchus*) aged PD7 (*n*=2; 0 male:2 female), PM1 (*n*=2; 1:1), PM3 (*n*=2; 1:1), PM9 (*n*=2; 0:2), PM12 (*n*=2; 1:1) and adult (>2.5 years; *n*=2; 1:1) were selected for this study. Gender was not a criterion in the selection of animals. Animals were procured from the National Non-human Primate Breeding and Research Facility (Australia) and housed in a vivarium (12:12 h light/dark cycle, temperature 31°C, humidity 65%). All experiments were conducted in accordance with the Australian Code of Practice for the Care and Use of Animals for Scientific Purposes and were approved by the Monash University Animal Ethics Committee, which also monitored the welfare of the animals.

### Surgical procedure

N-methyl-D-aspartate (NMDA) was administered bilaterally to the PM of 14- to 18-day-old marmoset monkeys (*n*=3; 2 male); a stage when thalamocortical circuits are immature. We have previously described the method in full (Mundinano et al., 2016). All procedures were performed under general anesthesia, and analgesics were provided post-surgery. In brief, the animal was placed in an MRI-compatible stereotaxic frame. Following craniotomies over each hemisphere, T2-weighted MRI scans were used to identify PM brain regions for stereotactic injections. NMDA (200 mM; 180 nl) was pressure-injected into the right and left PM regions at defined coordinates. The general health of the neonates, such as oxygen saturation and temperature, was monitored until full recovery and their return to the family unit. The animals were allowed >18 months to reach full maturity for subsequent analyses. The anatomical analysis, including SST^+^ NeuN^+^ neuronal cell counting, was performed in adulthood (*n*=3).

### Tissue processing

Animals were administered an overdose of pentobarbitone sodium (100 mg/kg). Following apnoea, neonates and juveniles were transcardially flushed with warm (∼30°C) heparinized phosphate buffer (PB) 0.1 M (pH 7.2) containing 0.1% sodium nitrite, and adults with room temperature heparinized saline (0.9%). All animals were subsequently perfused with 4% paraformaldehyde in PB 0.1 M and postfixed overnight in 4% paraformaldehyde at 4°C, dehydrated in increasing concentrations of sucrose (10, 20 and 30%) in PB 0.1 M, frozen over a bath of liquid nitrogen, and stored at −80°C until cryosectioning.

### Histology and immunolabeling

Each brain hemisphere was cut in the coronal plane on a cryostat (CM3050S; Leica) at a thickness of 50 µm, divided into four series, and stored free-floating in a cryoprotective solution [50% phosphate-buffered saline (PBS), 30% ethylene glycol, 20% glycerol] at −20°C. Sections were rinsed in PBS and blocked in a solution of PBS, 0.3% Triton X-100 and 5% normal donkey serum for 1 h at room temperature. Sections were incubated with the primary antibodies (see Table S1; combined in the case of double-labeling) and biotin-conjugated WFA (1:400; L1516, Sigma-Aldrich) for PNN labeling, diluted in the blocking solution for 16-18 h at 4°C. For SST/NeuN double-labeling, Triton X-100 was omitted from the blocking solution, and sections were incubated for 48-72 h at 4°C. Following incubation, sections were rinsed three times in PBS for 10 min each, incubated with the appropriate secondary antibodies in donkey anti-rabbit Alexa Fluor 488 (Thermo Fisher Scientific; RRID: AB_2535792; 1:800), goat anti-guinea pig Alexa Fluor 488 (Abcam; RRID: AB_2736871; 1:800), donkey anti-mouse Alexa Fluor 594 (Thermo Fisher Scientific; RRID: AB_2535789; 1:800), donkey anti-mouse Alexa Fluor 488 (Thermo Fisher Scientific; RRID: AB_141607; 1:800), donkey anti-rabbit Alexa Fluor 594 (Thermo Fisher Scientific; RRID: AB_141637; 1:800) and goat anti-rat Alexa Fluor 488 (Thermo Fisher Scientific; RRID: AB_253407; 1:800) secondary antibodies as well as streptavidin Alexa Fluor 594 (Thermo Fisher Scientific; A511227; 1:800) (Molecular Probes) in the blocking solution for 1 h at room temperature, rinsed three times in PBS for 10 min, and incubated with Hoechst 33258 (pentahydrate bis-benzimide; Dako, H1398) to visualize cell nuclei. The sections were then rinsed in PBS, mounted on Superfrost Plus glass slides (Thermo Fisher Scientific) with Fluoromount G mounting medium (Thermo Fisher Scientific) and coverslipped. The labeling was carried out with only the secondary antibody as a negative control.

### Microscopy

Sections were imaged with an Axio.Imager Z1 epifluorescence microscope (Zeiss) equipped with an Apotome to acquire near-confocal quality *z*-stacks. Three images of each section were obtained with a Zeiss Axiocam HRm digital camera using Axiovision software (v.4.8.1.0) at a resolution of 1024×1024 pixels, saved as Zeiss Vision Image (ZVI) and exported to tagged image file format (TIFF) format. The objectives used were Zeiss EC-Plan-Neofluar 5×0.16 (420330-9901), EC-Plan-Neofluar 10×0.3 (420340-9901) and Plan Apochromat 20×0.8 (420650-9901). Filter sets used for visualizing fluorescently labeled cells were Zeiss 49 4′,6-diamidino-2-phenylindole (DAPI) (488049-9901-000), Zeiss HE enhanced Green Fluorescent Protein (eGFP) (489038-9901-000) and Zeiss HQ Texas Red (000000-1114-462).

Low-magnification photomicrographs (1300×1030 dpi) were acquired with a Zeiss Discovery V20 stereomicroscope and an Axiocam HRc camera connected to Axiovision (v.4.7.1) (Zeiss Microscopy).

To confirm the colocalization of SST/NeuN, PV/NeuN and WFA/NeuN by double immunostainings, a *z*-stack was captured on a C1 inverted confocal microscope (Nikon, Tokyo, Japan). The microscope was equipped with excitation laser lines at 405, 488, 561 and 638 nm, and the images were obtained at a scan size of 1024×1024 bpi, with frame averaging set to 2. Two objectives, ×40 and ×60, were used. The acquired images were processed using NIS software (Nikon) and analyzed in Fiji (NIH).

### Digital image processing and quantification

Image stitching, and contrast and brightness adjustments were performed using Adobe Photoshop CC 2019. Figures, including contours, labels and annotations, were composed using Adobe Illustrator CC 2019.

For PV, SST and PNN labeling quantification, only cortical L3 and L5-6 were considered. These layers were selected because they act as input and output in the PFC. Hence, their maturation reflects neuronal network connectivity maturation in the PFC. For each animal, three images of each cortical area were randomly captured, and cell quantification was performed using the Fiji ‘Cell Counter’ plugin. The counting frame was estimated to be 440×340 µm^2^. The results are shown as a ratio of SST^+^ NeuN^+^ cells, PV^+^ NeuN^+^ cells and WFA^+^ PV^+^ cells. For each age, the fluorescence intensity of L3 and L5-6 was measured using Fiji image software (Schindelin et al., 2012), and the result was normalized to the fluorescence intensity of L1, which did not exhibit a signal and only emitted autofluorescence, often a result of aldehyde fixation. The ion channel fluorescence intensity was quantified in an average number of 20 cells by Fiji according to previously described methods (Akhter et al., 2019). Briefly, single-plane images (taken with a ×40 objective) with the largest surface area of PV^+^ cells were used for ion channel analysis. The PV^+^ cell membrane (mode: 8-connected; tolerance: 1000) was outlined automatically using wand tracing tool in Fiji. The trace was converted to a line of 0.5 μm width, and the average intensity of the ion channel signal convergent with the line was calculated (gray level, 12 bits). The values obtained were normalized to the autofluorescence signal in the background, obtained from a 10 µm^2^ square area in the same optical plane adjacent to the PV^+^ cells and lacking labeled neuropil. The results were presented as a percentage over the background using the following equation:

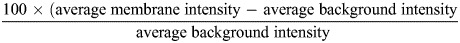
To predict the developmental stage at which PV^+^ INs exhibit fast-spiking action potential, the association between the fluorescence intensity of ion channels Kv3.1b and Nav1.1 was profiled across postnatal development using the ratio:

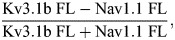
where FL represents fluorescence signal.

### Statistical analysis

Statistical analyses were performed using GraphPad Prism software (v.9.5.1). All data are presented as median±interquartile range. A *P-*value ≤0.05 was considered statistically significant. A nonparametric Kruskal–Wallis test followed by Dunn's posthoc test was applied to the cell count ratio and ion channel analysis data. While the data in the manuscript were presented as median±interquartile ranges, medians were used to represent data in figures unless stated otherwise.

A Mann–Whitney test compared the fluorescence intensity levels between L3 and L5-6 for all stages. The data are presented as median±interquartile ranges. A *P*-value <0.05 was considered significant. A nonparametric Kendall correlation test was performed to detect an association between Nav1.1 and Kv3.1b at the different developmental stages.

## Supplementary Material



10.1242/develop.204254_sup1 Supplementary information
