# Supplementary material for: Developmental dynamics of marmoset prefrontal cortical SST and PV interneuron networks highlight primate-specific features
Source: Development. 2025 May 19;152(10):dev204254. doi: 10.1242/dev.204254 (PMC12148029; doi:10.1242/dev.204254)
Supplement: Supplementary information [file develop-152-204254-s1.pdf]

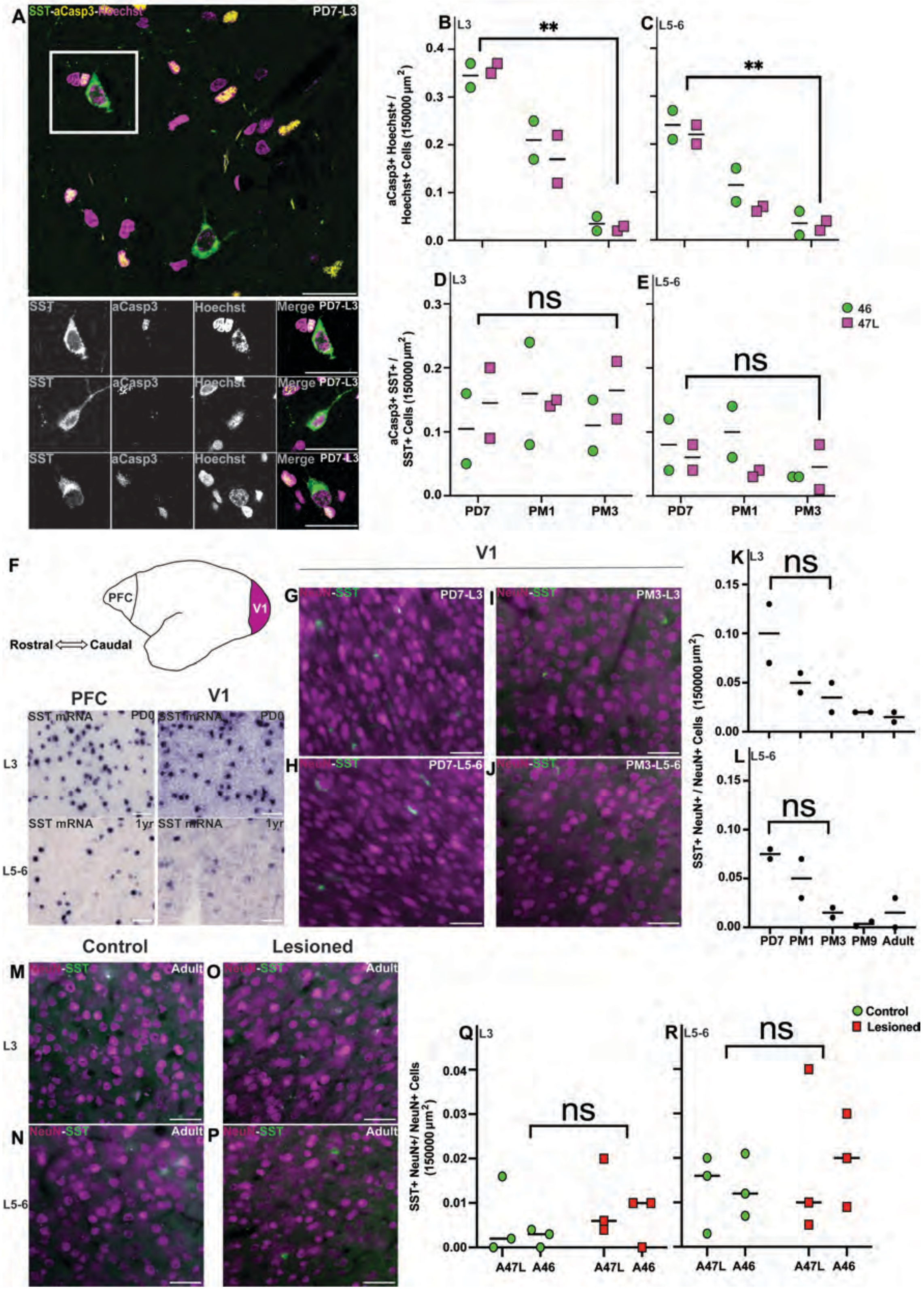


Development • Supplementary information

**Fig. S1. The decrease in the density of SST+ cells across development did not occur due to apoptosis.** **A** The images represent triple staining of SST (green), aCasp3 (yellow), and nucleus (magenta) at magnification 60x in L3 of areas 46 at the stages of PD7, PM1, and PM3. **B** and **C** The ratio of aCasp3+Hoechst+/ Hoechst+ cells were counted in L3 and 5/6 of areas 46 and 47L at a magnification of x20. The data analysis identified that developmental apoptosis reduced significantly from PD7 to PM3. **D** and **E** The density of aCasp3+SST+ cells was counted in L3 and 5/6 of areas 46 and 47L at a magnification of x20; the data analysis did not reveal a significant change in the population of aCasp3+SST+ interneurons in the studied areas suggesting that the postnatal decrease in the fraction of SST+ cells did not happen due to developmental apoptosis. **F** In situ hybridization data from the marmoset gene atlas illustrates the changes in SST mRNA in area 8aV and V1 at birth (PD0) and 1 year of age. Immunostaining represents SST (green)/ NeuN (magenta) double labeling in V1 at PD7 and PM3 at x20 magnifications (**G-J**). **K** and **L** The ratio of SST+NeuN+ INs over the total number of NeuN+ neurons per 150,000$\mu m^2$ was calculated at each developmental stage. The data analysis did not show a significant change in the faction between PD7 and PM3 in both L3 and L5-6 (n=2). Immunostaining represents SST (green)/ NeuN (magenta) double labeling in area 46 of the PM lesioned and control animals at x20 magnifications in adulthood (**M-P**). **Q** and **R** The data analysis did not show a significant change in the faction between PM lesioned and control animals in both L3 and L5-6 of areas 46 and 47L. The variability between the two studied areas was not also identified to be significant (n=3). For data analysis, Dunn's multiple comparisons accompanied the nonparametric Kruskal-Wallis test. The data were presented as median ± interquartile ranges, $p<0.05$: significant, ns: non-significant.

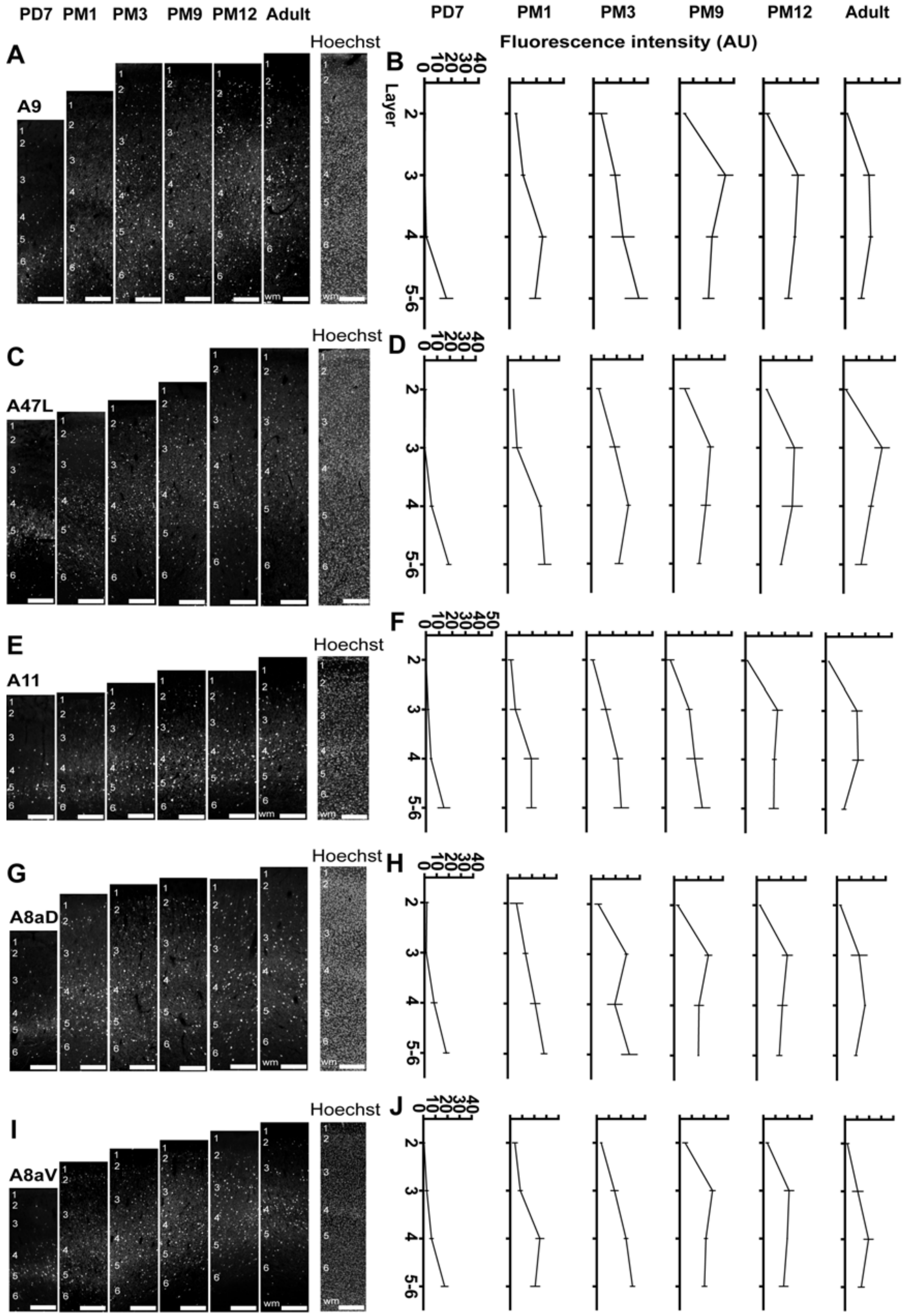


**Fig. S2. Consistent modifications of PV laminar distribution across prefrontal areas.**

**A, C, E, G,** and **I** The immunostainings show postnatal developmental modifications of PV (grey) across the laminae in areas 9, 47L, 11, 8aD, and 8aV, respectively. **B, D, F, H,** and **J** The graphs consistently show the quantifications of PV fluorescence intensity in areas 9, 47L, 11, 8aD, and 8aV, respectively. The populations of PV+ interneurons were initially observed in infragranular layers at PD7 and gradually increased towards supragranular L3.

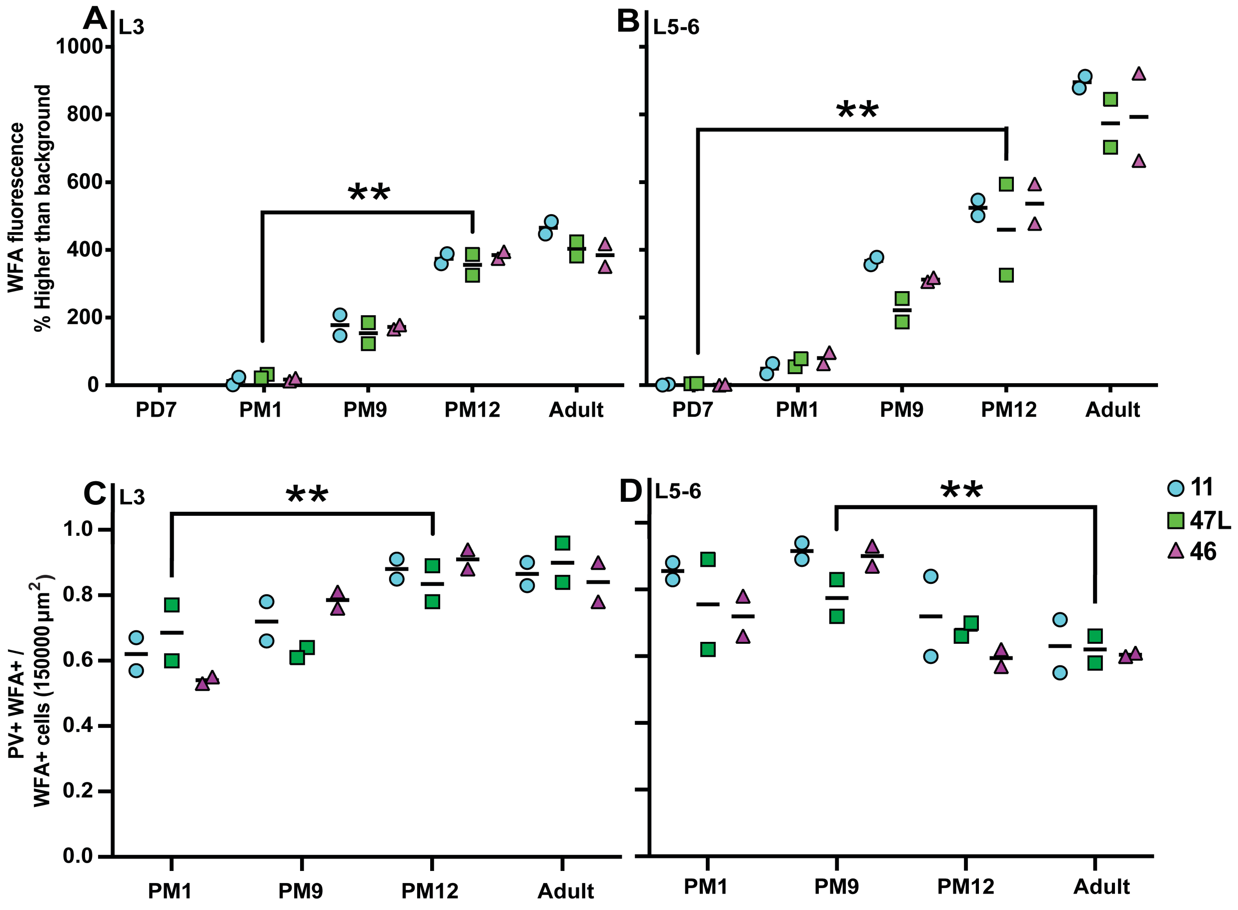


**Fig. S3. Consistent increase in PNN fluorescence intensity across prefrontal areas during mid-adolescence.**

**A** and **B** Graphs represent a gradual postnatal increase in the fluorescence intensity of WFA in the cell membrane of PV+ cells in L3 and 5/6 in areas 11, 47L, and 46 at PM12 correlated with mid-adolescence. The autofluorescence intensity of the background normalized the amounts of WFA FI. **C** and **D** The postnatal developmental modifications of the fraction of PV+WFA+/ WFA+ cells in L3 and 5/6 in areas 11, 47L, and 46. While there was a significant increase in the fraction between PM1 and PM12 of L3, a significant decrease in the population was identified in L5-6 between PM9 and adulthood. For data analysis, Dunn's multiple comparisons accompanied the nonparametric Kruskal-Wallis test. The data were presented as median ± interquartile ranges, * $p<0.05$: significant (n=2).

Development • Supplementary information

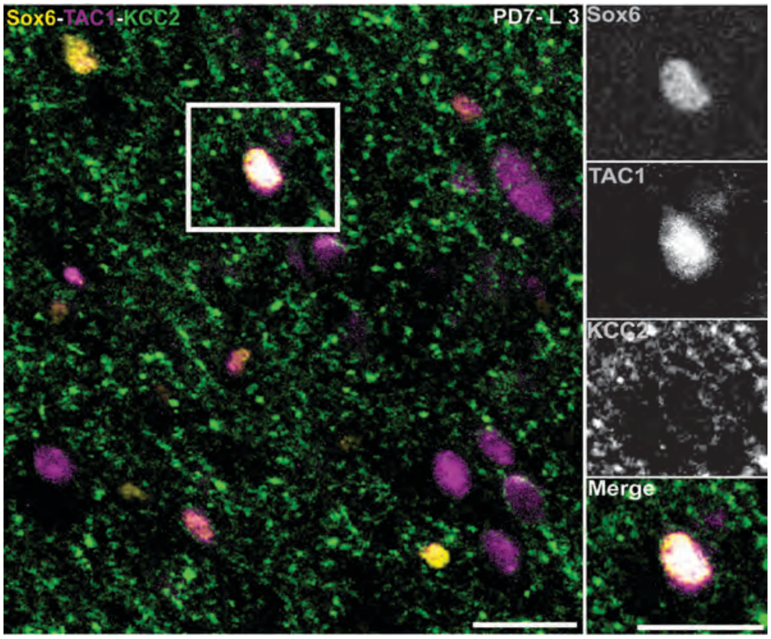


**Fig. S4. Expression of KCC2 in presumptive PV+**

Presumptive PV+ INs identified by Sox6 (yellow) and Tac1 (magenta) express KCC2 (green) in L3 of area 8a in neonates (PD7), suggesting electrical activity of precursors of PV+ cells before PV upregulation.

## Table S1. List of primary antibodies employed in the study

| Antigen | Abbreviation | Host Species | Dilution | Source | Catalog/clone | RRID |
|---|---|---|---|---|---|---|
| **Neuronal nuclear antigen, clone A60** | NeuN | Rabbit | 1:1000 | Merck | MAB377 | AB_2298772 |
| **Parvalbumin (mono/ poly)** | PV | Mouse and Rabbit | 1:1000 | Swant | PV28, PV235 | AB_2315235 AB_10000343 |
| **Somatostatin** | SST | Rat | 1:100 | Merck | MAB2358 | AB_2722572 |
| **Active Caspase 3** | aCasp3 | Rabbit | 1:200 | Thermo Fisher | MA5-32015 | AB_2809309 |
| **Potassium/Chloride cotransporter 2** | KCC2 | Rabbit | 1:150 | Cell Signaling Technology | 94725 | AB_2800232 |
| **Substance P** | Tac1 | Guinea pig | 1:500 | Boster Bio | A06666 | - |
| **Transcription factor** | Sox6 | Mouse | 1:200 | Thermo Fisher | MA5-31426 | AB_2787062 |
| **Potassium channel** | Kv3.1b | Rabbit | 1:500 | Merck | AB5188 | AB_91735 |
| **Sodium channel** | Nav1.1 | Rabbit | 1:200 | Alomone Labs | ASC-001 | AB_2040003 |
