# Supplementary material for: Developmental dynamics of marmoset prefrontal cortical SST and PV interneuron networks highlight primate-specific features
Source: Development. 2025 May 19;152(10):dev204254. doi: 10.1242/dev.204254 (PMC12148029; doi:10.1242/dev.204254)
Supplement: Supplementary file 1 [file develop-152-204254_review_history.pdf]

# Developmental dynamics of the marmoset prefrontal cortical SST and PV interneuron networks highlight primate-specific features

Nafiseh S. Hosseini Fin, Adrian Yip, Jack T. Scott, Leon Teo, Jihane Homman-Ludiye and James A. Bourne
DOI: 10.1242/dev.204254

**Editor**: Francois Guillemot

**Review timeline**

| | |
|---|---|
| Original submission: | 15 July 2024 |
| Editorial decision: | 23 August 2024 |
| First revision received: | 7 January 2025 |
| Editorial decision: | 28 January 2025 |
| Second revision received: | 17 April 2025 |
| Accepted: | 22 April 2025 |


---

**Original submission**

First decision letter

MS ID#: dev.204254
MS TITLE: Developmental dynamics of the prefrontal cortical SST and PV interneuron networks: Insights from the monkey highlight human-specific features

AUTHORS: Nafiseh Hosseini Fin; Adrian Yip; Leon Teo; Jihane Homman-Ludiye; James Bourne
ARTICLE TYPE: Research Article

Dear Dr Bourne,

I have now received all the referees' reports on the above manuscript, and have reached a decision. The referees' comments are appended below, or you can access them online: please go to:

As you will see, the referees express considerable interest in your work, but have some significant criticisms and recommend a substantial revision of your manuscript before we can consider publication. In particular, they recommend that you investigate whether the decline in numbers of Sst+ interneurons is specific to the prefrontal cortex or occurs across all areas of the marmoset cortex. If you are able to revise the manuscript along the lines suggested by the referees, which may involve further experiments, I will be happy to receive the revised version. Your revised paper will be re-reviewed the original referees, and acceptance of your manuscript will depend on your addressing satisfactorily their major concerns. Please also note that Development will normally permit only one round of major revision. If it would be helpful, you are welcome to contact us to discuss your revision in greater detail. Please send us a point-by-point response indicating your plans for addressing the referees' comments, and we will look over this and provide further guidance.

Please attend to all of the reviewers' comments and ensure that you clearly highlight all changes made in the revised manuscript. Please avoid using 'Tracked changes' in Word files as these are lost in PDF conversion. I should be grateful if you would also provide a point-by-point response detailing how you have dealt with the points raised by the reviewers in the 'Response to Reviewers' box. If you do not agree with any of their criticisms or suggestions please explain clearly why this is so.

© 2025. Published by The Company of Biologists under the terms of the Creative Commons Attribution License (https://creativecommons.org/licenses/by/4.0/).

## Reviewer 1

*Advance summary and potential significance to field*

In this paper, Fin et al. examined the developmental profile of medial ganglionic eminence-derived interneurons, PV+ and SST+ interneurons, in the developing marmoset brain. Most studies on developing MGE interneurons have been mostly done in rodent brains, this work provides a timely insight into how this class of interneurons develops in the primate brain.

In the first part, the developmental trajectory of SST+ interneurons was examined across different developmental stages of the marmoset brain. Similar to what has been discovered in rodents, SST interneurons are known to develop first compared to PV interneurons. However, unlike rodents, there is a sharp decline in the proportion of SST+ cells during development.

In the second part, the developmental trajectory of PV+ interneurons was examined. Here, the authors have described different developmental dynamics between PV interneurons in the infragranular layers and those in the supragranular layers. Here, not only have the authors examined the expression of PV, but they have also examined the expression of KCC2, KV3.1 and Nav1.1 in PV cells.

Altogether, although this work provides insights into the spatial-temporal dynamics of MGE interneuron development in the developing marmoset brain, additional clarification and queries need to be address before the authors can fully support their claims.

*Comments for the author*

Major points:

1. The authors claim that the decrease in the proportion of SST cells is linked to the downregulation of SST, especially since no difference in cleaved caspase 3+ SST+ cells was detected in the time window examined. There are several problems with this claim - 1) the time window in which a cell is positive for the cleaved caspase 3 is narrow (~1-2hours before cell death), consequently, the probability of capturing a positive cell is limited, 2) it has been shown that in rodents programmed cell death occurs within the first few weeks of mouse postnatal development (PMID: 23041929, 29849154). While this is unknown in primates, it is also likely if it happens, it could occur beyond the current resolution of the tissue sampled. To make this claim, the author should stain for a marker of all MGE interneurons (e.g. Lhx6). If the authors' claim is true, then there should be no difference between Lhx6+ cells in the adult brain as compared to the younger ages (e.g.PD7) but an increase in Lhx6+ SST- cells with development. If this experiment is not feasible, the authors should tone down their claim.

2. In all figures 2,3 and 5, it is worth showing the colocalised images of NeuN and the interneuron markers across all ages for one representative area (similar to Fig 4A). This will give a better sense of the data.

3. The authors have described that in the infragranular layer, the ratio of PV to NeuN cells remains relatively constant between PM9 and PM12. However, the images in Figure 4A suggest a marked decrease in PV cell density. The authors should explain this discrepancy.

4. The authors have linked the level of expression of PV cells to PV function. While this is not incorrect, the methodology used to measure PV intensity layer by layer is inappropriate. The authors should instead measure PV cell intensity from the cells directly. This will provide a better representation of the claim that the authors are trying to make. This can be easily done using tools such as CellProfiler (https://cellprofiler.org) which is high-throughput and free to use.

5. In Figure 5a, the image used does not seem to reflect the claim that the majority of PV cells are cloaked with WFA.

© 2025. Published by The Company of Biologists under the terms of the Creative Commons Attribution License (https://creativecommons.org/licenses/by/4.0/).

6. In Figure 6, the authors demonstrate that the KCC2 expression precedes PV expression. It is unclear what the authors are trying to claim here, especially since KCC2 is expressed in all MGE interneurons and is required for the termination of their migration (PMID:19376067). Hence, it is unsurprising that KCC2 expression is expressed before PV expression and may have nothing to do with PV expression. The authors should give a clearer explanation of the objectives of this experiment. If the authors are trying to demonstrate potential mechanisms driving PV maturation, perhaps staining for more PV maturation-specific mechanisms such as PGC1a is more useful (PMID: 20505089).

7. The authors should tone down this sentence

"The alterations observed, coupled with the evolutionary enlargement of the PFC, incorporating newly identified regions, many featuring a granule cell layer, provide substantive evidence endorsing our hypothesis that the developmental progression of inhibitory neurons (INs) within the primate PFC postnatally follows distinctive mechanisms compared to rodents."

While this may be true in some instances such as the SST, most of the things described in this paper are also observed in rodents.

Minor points:

1. Some rephrasing is required on the methodology described for the quantification of interneurons in Fig 2. The authors have mentioned that they have normalised the number of SST interneurons to the total number of neurons. But based on the methodology described, it seems to be the total number of neurons in a given area.

2. The author should rephrase this sentence
"The main factor determining the timing of maturation appears to be the IN laminar identity, as we consistently observed that the infragranular layer exhibited evidence of maturation before their supragranular counterparts"
Maturation is tied to birth date rather than based on the layer they terminate in.

3. While it is true that in primates there is an increase in interneurons present in the cerebral cortex. Most of this increase is due to an evolutionary increase in calretinin+ cells. (PMID:25018702). While this does not negate the importance of this work, it is misleading to the reader and the authors should clarify this point.

4. The choice of graph used to represent the quantifications can be at hard to follow and too crowded (especially Figures 6 and 7). The authors should consider changing the graphs so that it is easier for the readers to understand the graph.

**Reviewer 2**

*Advance summary and potential significance to field*

This manuscript presents a detailed analysis of the molecular profiles of interneurons in the developing PFC of marmosets. Understanding the development of GABAergic interneuron subtypes in the cognitive brain regions of primates is crucial. The findings are well-presented and include intriguing observations, such as the decline in the Sst interneuron population during the early postnatal adolescent period. Having detailed interneuron profiles at specific developmental time points is extremely valuable to the field.

*Comments for the author*

The strength of this study lies in the authors' first-time description of the developmental expression of interneuron markers in the PFC of marmosets. For example, Calb2 shows different patterns in rats and mice; it is not co-expressed in Sst cells in rats but is co-expressed in mice. This discrepancy initially caused confusion in characterizing the developmental origins of mouse interneuron classes. Since this is the first study of its kind in marmosets, a more careful distinction

© 2025. Published by The Company of Biologists under the terms of the Creative Commons Attribution License (https://creativecommons.org/licenses/by/4.0/).

between Pvalb and Sst interneuron classes is important.Characteristic features of interneuron subtypes are not solely determined by molecular profiles. Laminar locations, morphologies, and intrinsic firing properties are often used to characterize interneuron classes. While the observed decrease in Sst interneurons over time is unexpected, the increases in Pvalb expression, perineuronal net-related WFA, KCC2, and Kv and Nav channels align with anticipated developmental changes. Since the decline in Sst interneuron numbers is particularly interesting, it would be valuable to explore this in the main results. Is this phenomenon specific to the PFC area, or is it a general process across all cortical areas of the marmoset? Can you manipulate the circuit to determine if the decline in Sst interneuron numbers is linked to any specific developmental event? For example, if the reduction in Sst cells is also observed in the visual cortex, what happens when dark rearing or altering the eye field on one side? Overall, the current version lacks mechanistic insights to explain the observed decrease in Sst interneurons or the increase in the proteins of Pvalb and others during development. These issues should be addressed before the manuscript can be considered for publication in Development. Minor points for consideration are also included below.
Figure 1:
* In the results section, first paragraph: Please reference the panels in Figure 1C showing the Hoechst staining, and provide an explanation of the NNF stains in the context of the reference.
Figure 2:
* The Y-axis label "150,000 $\mu m^2$" is confusing and should be removed. Instead, label the axis with "SST+NeuN+/NeuN+". The current labeling is misleading and suggests that cell numbers are being counted per surface area. This adjustment should be applied to the remaining other figures as well.
Figure 3:
* It has been demonstrated in rodent models that Pvalb expression is activity-dependent; for example, Pvalb expression in the primary visual cortex is nearly abolished when mice are dark-reared. Given this, the phrase "increase in the proportion of PV+ interneurons" may be misleading. It might be more accurate to describe it as an "increase in Pvalb expression within interneurons."
* If the authors wish to explore this point further, it is strongly recommended to perform double in situ hybridization for Gad1/Pvalb, Lhx6/Pvalb, or Sox6/Pvalb at multiple developmental time points. This strategy will allow authors to resolve the issues related to Pvalb cell numbers vs. Pvalb expression onset.
* With the current data showing PV+NeuN+ double-positive cells relative to NeuN+ numbers, it is not possible to conclusively determine an increase in Pvalb+ interneuron cell numbers. The data may instead reflect the onset of Pvalb expression.
Figure 4:
* The argument presented for Figure 3 also applies here.
Figure 5:
* Modify the Y-axis label to read "PV+WFA+/PV+." Additionally, please provide data to show PV+WFA+/WFA+.
Figures 6 and 7:
* The analyses presented are well-conducted. However, it is important to note that the analysis of PV expression does not necessarily reflect the analysis of PV lineage cells. The authors are examining relatively mature PV+ populations, rather than comparing the entire population of PV+ cells with KCC2, Kv3.1, or Nav1.1.

---

**First revision**

***<u>Author’s response to the reviewer’s comments</u>***

| Comment | Response |
|---|---|
| **Reviewer 1** | |
| 1. The authors claim that the decrease in the proportion of SST cells is linked to the downregulation of SST, especially since no difference in cleaved caspase 3+ SST+ cells | We included following experiments related to SST results (Figure S1) for more clarification; First, the fraction of aCasp3+ Hoechst+ cells was quantified from PD7 to PM3 in the marmoset PFC. |

© 2025. Published by The Company of Biologists under the terms of the Creative Commons Attribution License (https://creativecommons.org/licenses/by/4.0/).

| | |
|---|---|
| was detected in the time window examined. There are several problems with this claim - 1) the time window in which a cell is positive for the cleaved caspase 3 is narrow (~1-2hours before cell death), consequently, the probability of capturing a positive cell is limited, 2) it has been shown that in rodents programmed cell death occurs within the first few weeks of mouse postnatal development (PMID: 23041929, 29849154). While this is unknown in primates, it is also likely if it happens, it could occur beyond the current resolution of the tissue sampled. To make this claim, the author should stain for a marker of all MGE interneurons (e.g. Lhx6). If the authors' claim is true, then there should be no difference between Lhx6+ cells in the adult brain as compared to the younger ages (e.g.PD7) but an increase in Lhx6+ SST- cells with development. If this experiment is not feasible, the authors should tone down their claim. | The results represented highest aCasp3+ Hoechst+ cells at PD7, which decreased by PM3 (S1, B and C). This contrasts with our observations in consistency of fraction of SST+ aCasp3+ cells between these stages (S1, D and E), suggesting that apoptosis may not account for the developmental reduction of majority of SST+ interneurons between these stages.<br><br>In addition, using *in situ* hybridisation data available online in Marmoset Gene Atlas, we illustrated *in situ* hybridisation data of SST mRNA in area 8aV of the PFC and primary visual area (V1) of the visual cortex in PD0 (birth) and 1 year of age (S1, F). In harmony with the SST+ cell countin4g data presented in the paper (Fig. 2), SST mRNA appears to reduce from birth until adulthood in these regions. To test the hypothesis in V1, we quantified SST+ NeuN+ cells in the marmoset V1 across postnatal development (S1, G-L). While there appeared to be a developmental reduction in the SST+ NeuN+ cells in V1, it was not statistically significant (S1, K and L), which is in contrast with our earlier observations for the PFC (Fig. 2) supporting the spatiotemporal variability of SST profile in the marmoset cortex. |
| 2. In all figures 2,3 and 5, it is worth showing the colocalised images of NeuN and the interneuron markers across all ages for one representative area (like Fig 4A). This will give a better sense of the data. | Figures 2, 3 and 5 A, already included a representative PFC strip of interneuron marker and NeuN for illustration. We only included developmental strips of PV marker at all ages since this interneuron was the focus of majority of experiments across the paper and PV marker changes in the laminae was also quantified in all ages (Figure 4). |
| 3. The authors have described that in the infragranular layer, the ratio of PV to NeuN cells remains relatively constant between PM9 and PM12. However, the images in Figure 4A suggest a marked decrease in PV cell density. The authors should explain this discrepancy. | We have replaced the representative PV strip of PM9. Please note that the cell density graph in Figure 3H and PV strips in Figure 4A represents two different experiments. Figure 3H represents gradual decrease in the density of PV+ NeuN+ cells in the infragranular layers between PM3 and PM12. However, Figure 4A and graph in Figure 4C are representative of PV marker intensity. Thus, the difference may result from the fact that each represents distinct features of density and intensity, which is statistically comparative. Second, sampling variability (in Figure FA) may cause a difference in this image which is not statistically significant as quantified in figure 4C. |
| 4. The authors have linked the level of expression of PV cells to PV function. While this is not incorrect, the methodology used to measure PV intensity layer by layer is inappropriate. The authors should instead measure PV cell intensity from the cells directly. This will provide a better representation of the claim that the authors are trying to make. This can be easily done | We respectfully disagree with your statement; PV is not only expressed in cell body but also expressed in the processes of PV+ interneurons including dendritic arbours, indicating their level of ramification and connectivity. Once PV+ interneurons fully mature they establish a network of connectivity to perform their specialised functions. Hence, we aimed to measure PV intensity not only in cell body but |

© 2025. Published by The Company of Biologists under the terms of the Creative Commons Attribution License (https://creativecommons.org/licenses/by/4.0/).

| | |
|---|---|
| using tools such as CellProfiler (https://cellprofiler.org) which is high-throughput and free to use. | also their processes in the laminae. The used method provides more insights into the changes in PV expression in the PFC during the maturation process. |
| 5. In Figure 5a, the image used does not seem to reflect the claim that the majority of PV cells are cloaked with WFA. | The contrast of this image has been adjusted for greater clarity. Please note that the majority of mature PV+ interneurons are indeed enwrapped by CPSGs, but the level of CPSG deposition may vary between cells, which may result in a difficulty to spot WFA in low magnification PV+WFA+ strip. |
| 6. In Figure 6, the authors demonstrate that the KCC2 expression precedes PV expression. It is unclear what the authors are trying to claim here, especially since KCC2 is expressed in all MGE interneurons and is required for the termination of their migration (PMID:19376067). Hence, it is unsurprising that KCC2 expression is expressed before PV expression and may have nothing to do with PV expression. The authors should give a clearer explanation of the objectives of this experiment. If the authors are trying to demonstrate potential mechanisms driving PV maturation, perhaps staining for more PV maturation-specific mechanisms such as PGC1a is more useful (PMID: 20505089). | The concept of KCC2 function in termination of interneuron migration is irrelevant to the aim of this experiment. First, the majority of MGE derived cells are PV+ interneurons as mentioned in your reference (PMID:19376067), second, we used specific marker of prospective PV+ cells to exclude other MGE derived interneurons (Sox6+ Tac1+ cells). It is previously hypothesised that upregulation of PV is linked to neuronal activity. While this hypothesis is investigated in diseased conditions (such as epilepsy), there is no direct evidence that suggest neuronal activity derives early developmental PV upregulation, so a primary question was to test whether the neuronal activity (regulated by KCC2 expression) is present before the upregulation of PV in prospective PV+ interneurons. Thus, this experiment provides indirect anatomical evidence regarding the presence of neuronal activity in prospective PV+ interneurons while the likely source(s) of this activity in the PFC needs further experimentations. Further explanations were added to the manuscript on Page 16. |
| 7. The authors should tone down this sentence<br><br>"The alterations observed, coupled with the evolutionary enlargement of the PFC, incorporating newly identified regions, many featuring a granule cell layer, provide substantive evidence endorsing our hypothesis that the developmental progression of inhibitory neurons (INs) within the primate PFC postnatally follows distinctive mechanisms compared to rodents."<br><br>While this may be true in some instances such as the SST, most of the things described in this paper are also observed in rodents. | Consistent maturation pattern of granular prefrontal areas is not studied or observed in rodents, since the cortices examined in this study (such as DLPFC) are primate specific. Further, a hierarchical pattern of marmoset visual cortex maturation was previously demonstrated using maturation markers [1], which is in contrast with data presented here in the PFC. Hence, this statement points out underlying mechanism(s) of maturation of MGE derived neurons in granular prefrontal areas may be distinct compared to their counterparts in rodents as well as primate sensory cortex. However, changes were made to the statement which now reads: “The observations in the present study, coupled with the evolutionary enlargement of the PFC, incorporating newly identified regions, many featuring a granule cell layer, highlights that the developmental progression of INs within the primate granular PFC postnatally may follow a distinctive mechanism compared to rodents as well as primate sensory cortex” (P20). |

© 2025. Published by The Company of Biologists under the terms of the Creative Commons Attribution License (https://creativecommons.org/licenses/by/4.0/).

| | |
|---|---|
| **Minor points:**<br><br>1. Some rephrasing is required on the methodology described for the quantification of interneurons in Fig 2. The authors have mentioned that they have normalised the number of SST interneurons to the total number of neurons. But based on the methodology described, it seems to be the total number of neurons in a given area. | Apologies, we meant total number of neurons in each counting frame, not area! Revision was made for further clarification, and it now reads: “We normalized our values to the total number of neurons in each counting frame to account for cortical expansion due to the addition of glial cells and synapses during postnatal development.” |
| 2. The author should rephrase this sentence "The main factor determining the timing of maturation appears to be the IN laminar identity, as we consistently observed that the infragranular layer exhibited evidence of maturation before their supragranular counterparts"<br>Maturation is tied to birth date rather than based on the layer they terminate in. | We rephrased this statement to be specific about PV INs and it now reads:” The main factor determining the timing of maturation of PV INs appears to be their laminar identity, as we consistently observed that the infragranular layer exhibited evidence of maturation before their supragranular counterparts” (P22).<br><br>Please note that the maturation is not necessarily tied to birthdate (unlike the inside-out pattern of maturation observed in excitatory neurons) for example, CGE derived interneurons which are generated later than MGE derived interneurons predominantly occupy superficial cortical layers, regardless of their birthdate [2, 3]. |
| 3. While it is true that in primates there is an increase in interneurons present in the cerebral cortex. Most of this increase is due to an evolutionary increase in calretinin+ cells. (PMID:25018702). While this does not negate the importance of this work, it is misleading to the reader and the authors should clarify this point. | Considering the paper statement of “While INs account for 15–20 % of total cortical neurons in rodents, this ratio soars to 25–34% in primates [4, 5]” we understand your statement regarding the increase in CGE derived interneurons in primates compared to rodents. However, here we just meant to represent the general differences in interneuron population between primate and rodents, especially for the reader that may not be well versed with the subject area. At this point of Introduction, we have not gone through MGE derived interneurons yet.<br><br>Please note that MGE derived interneurons have gone through a higher range of evolutionary diversity compared to CGE derived interneurons in primates than rodents [6] suggesting a higher variety of their functions in primates. This point was added to the statement for more clarification on differences in primate and rodents as it reads: “While INs account for 15–20 % of total cortical neurons in rodents, this ratio soars to 25–34% in primates. This expansion is accompanied by the evolutional diversification of interneurons, enabling a greater variety of functions in primates” (P3). |
| 4. The choice of graph used to represent the quantifications can be at hard to follow and too crowded (especially Figures 6 and 7). The authors should consider changing the graphs so that it is easier for the readers to understand the graph. | We revised the graphs in Figures 6 and 7, so they are now larger in size and more standout. |
| **Reviewer 2** | |

© 2025. Published by The Company of Biologists under the terms of the Creative Commons Attribution License (https://creativecommons.org/licenses/by/4.0/).

| | |
|---|---|
| The strength of this study lies in the authors' first-time description of the developmental expression of interneuron markers in the PFC of marmosets. For example, Calb2 shows different patterns in rats and mice; it is not co-expressed in SST cells in rats but is co-expressed in mice. This discrepancy initially caused confusion in characterizing the developmental origins of mouse interneuron classes. Since this is the first study of its kind in marmosets, a more careful distinction between Pvalb and Sst interneuron classes is important. Characteristic features of interneuron subtypes are not solely determined by molecular profiles. Laminar locations, morphologies, and intrinsic firing properties are often used to characterize interneuron classes. While the observed decrease in Sst interneurons over time is unexpected, the increases in Pvalb expression, perineuronal net-related WFA, KCC2, and Kv and Nav channels align with anticipated developmental changes. Since the decline in Sst interneuron numbers is particularly interesting, it would be valuable to explore this in the main results. Is this phenomenon specific to the PFC area, or is it a general process across all cortical areas of the marmoset? Can you manipulate the circuit to determine if the decline in Sst interneuron numbers is linked to any specific developmental event? For example, if the reduction in Sst cells is also observed in the visual cortex, what happens when dark rearing or altering the eye field on one side? Overall, the current version lacks mechanistic insights to explain the observed decrease in Sst interneurons or the increase in the proteins of Pvalb and others during development. These issues should be addressed before the manuscript can be considered for publication in Development. Minor points for consideration are also included below. | We appreciate your comments on this matter, and have included further experiments for more clarifications on this section:<br>1. to address whether the reduction of SST+ interneurons is reflected in other cortical areas, we performed SST NeuN immunostaining in the marmoset visual cortex (V1) and counted the fraction of SST+ NeuN+ interneurons in V1. Please see the response to major comment 1 of Reviewer 1 for more information.<br><br>2. to address whether developmental decline in the population of SST+ interneurons is linked to a developmental event, we have examined the frontal cortex of adult marmosets following a lesion of the medial pulvinar (PM) marmosets at a neonatal stage (postnatal day 14). PM is a primate specific higher order thalamic structure which establish a dense network of reciprocal connectivity with numerous associative cortical areas especially the PFC. The exact function of this connectivity is not well studied but it is presumed to play a modulatory role and be involved in directing selective attention. We observed the neonatal manipulation of this region is linked to a decrease of density of PV+ interneurons as well as cognitive dysfunctions related to PFC-PM circuit, but not in adults who received an identical lesion – suggestive of an early-life thalamic control of PV expression in the PFC (data not published here but is in preparation for another publication). In the present study, we investigated if the fraction of SST+ interneurons is altered in adult PM-lesioned animals. The SST+ NeuN+ counting results in the PFC of adult lesioned animals (areas 47L and 46) did not support a change in the number of SST+ NeuN+ cells in the PM lesioned group compared to the control (S1, M-R) suggesting that the developmental decline in SST+ cells is not related to the development and maturation of PM thalamic connectivity network with PFC. Based on these results, we provided mechanistic insights into observations related to SST+ cells changes during the postnatal development. |
| **Minor points:**<br><br>Figure 1:<br><br>* In the results section, first paragraph: Please reference the panels in Figure 1C | Figure 1C is explained in third paragraph of the Result section. The manuscript was revised now reads: “In short, variations in the evidence and thickness of L4 were the main point of reference for resolving the boundary between adjacent areas across all stages using Hoechst staining |

© 2025. Published by The Company of Biologists under the terms of the Creative Commons Attribution License (https://creativecommons.org/licenses/by/4.0/).

| | |
|---|---|
| showing the Hoechst staining and provide an explanation of the NNF stains in the context of the reference. | (Fig.1, C)."<br><br>The text was updated as it reads: "The NNF deposition in the basal and apical dendrites of the large pyramidal neurons consolidates the dendritic morphology [7]". (P7) |
| Figure 2:<br><br>* The Y-axis label "150,000 μm²" is confusing and should be removed. Instead, label the axis with "SST+NeuN+/NeuN+". The current labeling is misleading and suggests that cell numbers are being counted per surface area. This adjustment should be applied to the remaining other figures as well. | Cell numbers are indeed being counted per surface area (frame) (150000 μm²), and that is why this forms part of the graph labelling. We don't feel this should be removed as this provides context to the value on the Y-axis. |
| Figure 3:<br><br>* It has been demonstrated in rodent models that Pvalb expression is activity-dependent; for example, Pvalb expression in the primary visual cortex is nearly abolished when mice are dark-reared. Given this, the phrase "increase in the proportion of PV+ interneurons" may be misleading. It might be more accurate to describe it as an "increase in Pvalb expression within interneurons." If the authors wish to explore this point further, it is strongly recommended to perform double in situ hybridization for Gad1/Pvalb, Lhx6/Pvalb, or Sox6/Pvalb at multiple developmental time points. This strategy will allow authors to resolve the issues related to Pvalb cell numbers vs. Pvalb expression onset. | Here we used PV+ cell quantifications as an indication of maturation of these cells using PV as a maturation marker of a particular area. *In situ* hybridisation does not reflect PV protein expression. The only precise approach for PV protein expression is western blotting, however, if we used western blotting it would have limited our spatial analyses (e.g. layers/ cells). So, while we counted PV+ interneuron numbers to track PV expression across the postnatal development of the PFC areas in supra- and infra- granular layers, we believe it is more precise to represent the data as "increase in the proportion of PV+ interneurons" instead of increase in Pvalb expression within interneurons, as this better represents local circuit and area dynamics. |
| * With the current data showing PV+NeuN+ double-positive cells relative to NeuN+ numbers, it is not possible to conclusively determine an increase in Pvalb+ interneuron cell numbers. The data may instead reflect the onset of Pvalb expression. | We are unclear why our counting method, which is a common approach by many, is leading to confusion. We have counted the density of PV+ NeuN+ cells and presented as the fraction of this subtype to total neuronal cells, to exclude the effect of changes in neuronal cells across layer, age, or area. Also, as mentioned in previous response, the quantification of protein expression using western blotting wasn't necessarily appropriate method in this specific study. As complementary to cell counting data, in Figure 4, we also assessed PV expression by quantifying PV marker intensity across layers. |
| Figure 4:<br><br>* The argument presented for Figure 3 also applies here. | Please see the response to comment for Figure 3. |
| Figure 5:<br><br>* Modify the Y-axis label to read "PV+WFA+/PV+." Additionally, please provide data to show PV+WFA+/WFA+. | For the first part of the comment, please see my response to Figure 2 comment. We also quantified PV+WFA+/WFA+ in the PFC as suggested. Please see Figure S3 (C and D) for representation of this data. The manuscript is also updated and now reads: "To investigate the modifications of fraction of PV+WFA+ cells among WFA+ cells, the |

© 2025. Published by The Company of Biologists under the terms of the Creative Commons Attribution License (https://creativecommons.org/licenses/by/4.0/).

| | |
|---|---|
| | ratio of PV+WFA+ / WFA+ cells was quantified in the PFC areas 11, 47L and 46 across the postnatal development (Sup. Fig. S3, C and D). We detected a gradual increase in the population of PV+WFA+/ WFA+ cells from PM1 until PM12 (0.59 ± 0.15 vs. 0.88 ± 0.1, p=0.004) in layer 3 of the studied areas (Sup. Fig. S3, C), correlated with postnatal increase in the population of PV+ INs (Fig. 3, G) and PV+WFA+ cells (Fig 5, G) observed previously. A distinct pattern emerged in L5–6; the fraction of PV+WFA+/ WFA+ cells remained stable from PM1 to PM9 (p>0.05) (Sup. Fig. S3, D) supporting an earlier maturation of PV+ INs in infragranular layers (Fig. 3, H). Interestingly, this population declined from PM9 to adulthood (0.88 ± 0.13 vs. 0.61 ± 0.1, p=0.006) (Sup. Fig. S3, D) that possibly reflect the delayed maturation of **other neuronal subtypes** though PNNs deposition mechanism". (P14) |
| Figures 6 and 7:<br><br>* The analyses presented are well-conducted. However, it is important to note that the analysis of PV expression does not necessarily reflect the analysis of PV lineage cells. The authors are examining relatively mature PV+ populations, rather than comparing the entire population of PV+ cells with KCC2, Kv3.1, or Nav1.1. | Yes, we indeed assessed ion channel expressed in the cell membrane of PV+ interneurons across postnatal development. We used the markers of prospective PV+ cells at PD7 during which no PV expression was detected to be present in layer 3 (S4). |

---

<u>**Second decision letter**</u>

MS ID#: dev.204254R1

MS TITLE: Developmental dynamics of the prefrontal cortical SST and PV interneuron networks: Insights from the monkey highlight human-specific features

AUTHORS: Nafiseh Hosseini Fin; Adrian Yip; Leon Teo; Jack Scott; Jihane Homman-Ludiye; James Bourne

Dear Dr Bourne,

I have now received all the referees reports on the above manuscript, and have reached a decision. The referees' comments are appended below, or you can access them online: please go to .

The overall evaluation is positive and we would like to publish a revised manuscript in Development, provided that the referees' comments can be satisfactorily addressed. Please attend to all of the reviewers' comments in your revised manuscript and detail them in your point-by-point response. If you do not agree with any of their criticisms or suggestions explain clearly why this is so. If it would be helpful, you are welcome to contact us to discuss your revision in greater detail. Please send us a point-by-point response indicating your plans for addressing the referees' comments, and we will look over this and provide further guidance.

<u>Reviewer 1</u>

*Advance summary and potential significance to field*

© 2025. Published by The Company of Biologists under the terms of the Creative Commons Attribution License (https://creativecommons.org/licenses/by/4.0/). 

In this paper, Fin et al. examined the developmental profile of medial ganglionic eminence-derived interneurons, PV+ and SST+ interneurons, in the developing marmoset brain. Most studies on developing MGE interneurons have been mostly done in rodent brains, this work provides a timely insight into how this class of interneurons develops in the primate brain.

In the first part, the developmental trajectory of SST+ interneurons was examined across different developmental stages of the marmoset brain. Similar to what has been discovered in rodents, SST interneurons are known to develop first compared to PV interneurons. However, unlike rodents, there is a sharp decline in the proportion of SST+ cells during development. Here the authors have also demonstrated that the developmental deciline in SST interneurons are not related to the maturation of the medial pulvinar.

In the second part, the developmental trajectory of PV+ interneurons was examined. Here, the authors have described different developmental dynamics between PV interneurons in the infragranular layers and those in the supragranular layers. Here, not only have the authors examined the expression of PV, but they have also examined the expression of KCC2, KV3.1 and Nav1.1 in PV cells.

*Comments for the author*

Thank you to the authors for making changes in the original manuscript.
There are however two points that I would like to comment.

1) Just a minor comment. With regards to the manipulations using the medial pulivnar lesioned marmoset, the wording in the results section needs to be more carefully written. Just to avoid the misconception with the timing when the lesions are being done, phrases such as "adult PM-lesioned animals" and "adult lesioned animals" can be a little confusing, especially when the timing of when the lesions are done (which is done during neonatal period) play an important factor in your claim.

2) While the authors have a point that PV is expresesd in the dendritic arbor, what the author claims that measuring PV intensity would give a better insight into their maturation process is only true if PV interneurons are the only cells that express parvalbumin in the cortex. A subset of L5 pyramidal cells have been shown to expressed parvalbumin which in turn might overall impact laminar intensity. Hence, the authors should either 1) acknowledge that the PV intensity can also be contributed by these pyramidal cells or 2) that there are no PV-expressing pyramidal cells in the PFC at the time examined.

<u>Reviewer 2</u>

*Advance summary and potential significance to field*

The authors have improved the manuscript by adding data and rewriting the text. As previously stated, the strength of this study lies in the authors' first-time description of the developmental expression of interneuron markers in the PFC of marmosets. The gradual increase in Pvalb can be simply explained by the maturation of this cell type, and it is well-established that Pvalb is an activity-dependent gene in rodent circuits. The observed decline in Sst expression is intriguing; however, the current manuscript does not clearly distinguish whether this represents a reduction in Sst expression levels or a decrease in the number of Sst+ cells, despite addressing both protein and RNA levels of Sst. Reviewer 1 highlights the importance of demonstrating a marker for MGE-lineage interneurons, such as Lhx6, and suggests that if this is not possible, the authors should tone down the claim regarding a reduction in Sst+ cell numbers. I agree with this recommendation. A decrease in Sst interneurons during development is a significant claim, and I am not aware of similar observations in other cortical regions or animal models. If apoptosis (Figure S1) is not the cause, what mechanism explains the decrease in Sst+ cells? By utilizing MGE-lineage markers such as Lhx6 and Sox6, or even the pan-GABAergic marker Gad, the authors could clarify whether the observed decline reflects reduced Sst expression (in which case Lhx6 cell numbers would remain unchanged) or a decrease in Sst+ cell numbers (in which case Lhx6 levels would also decrease). Additionally, the manuscript would benefit from a plausible explanation for why Sst expression or Sst+ cell

© 2025. Published by The Company of Biologists under the terms of the Creative Commons Attribution License (https://creativecommons.org/licenses/by/4.0/).

numbers decrease in the juvenile PFC of marmosets. Similarly to what I have stated in the original review, the revised version still lacks mechanistic insights to explain the observed decrease in Sst interneurons or the increase in the proteins of Pvalb and others during development. These issues should be addressed before the manuscript can be considered for publication in Development.

Responses to the Original Major Points
1. It is reassuring to see that the decline in Sst expression is specific to the PFC and not observed in V1 (Figure S1).
2. The statement that "the developmental decline in Sst+ cells is not related to the development and maturation of PM thalamic connectivity networks with the PFC" does not provide mechanistic insights into why Sst+ cell numbers decrease during postnatal development. While I understand that manipulations in marmosets are challenging, if apoptosis (Figure S1) is ruled out, a reasonable explanation for this phenomenon remains necessary.

---

**Second revision**

Author response to reviewers' comments

**POINT-TO-POINT REBUTTAL**

**Reviewer 1:** SUMMARY OF THE ADVANCE MADE IN THIS PAPER AND ITS POTENTIAL SIGNIFICANCE TO THE FIELD

SUGGESTIONS TO AUTHORS

Thank you to the authors for making changes in the original manuscript.
There are however two points that I would like to comment.

1) Just a minor comment. With regards to the manipulations using the medial pulvinar lesioned marmoset, the wording in the results section needs to be more carefully written. Just to avoid the misconception with the timing when the lesions are being done, phrases such as "adult PM-lesioned animals" and "adult lesioned animals" can be a little confusing, especially when the timing of when the lesions are done (which is done during neonatal period) play an important factor in your claim.

We agree with Reviewer 1 that our nomenclature was confusing, and we updated the sentences as it reads:

” Here, we investigated if the fraction of SST+ INs is altered in adult animals (>2.5years) who received a lesion of PM neonatally (referred hereafter as lesioned animals) (Ln 267-268)

“ To address whether the decline in the population of SST+ INs is linked to a developmental event, we used PM thalamic lesioned marmoset models, developed in the Bourne laboratory (Mundinano, Flecknell, and Bourne 2016), in which the PM was ablated at neonatal stage (postnatal day 14-18) (Mundinano, Flecknell, and Bourne 2016) (Ln 256-258)

2) While the authors have a point that PV is expressed in the dendritic arbor, what the author claims that measuring PV intensity would give a better insight into their maturation process is only true if PV interneurons are the only cells that express parvalbumin in the cortex. A subset of L5 pyramidal cells have been shown to expressed parvalbumin which in turn might overall impact laminar intensity. Hence, the authors should either 1) acknowledge that the PV intensity can also be contributed by these pyramidal cells or 2) that there are no PV-expressing pyramidal cells in the PFC at the time examined.

Reviewer 1 makes an excellent point. We acknowledge previous observations indicating that some pyramidal neurons in the inner layers of the primate motor, somatosensory cortex, and primary visual area possess PV immunoreactivity (Preuss and Kaas 1996; Spatz, Illing, and Weisenhorn 1994a). However, our observation in the PFC has not revealed the 10~20% of pyramid-like PV+

© 2025. Published by The Company of Biologists under the terms of the Creative Commons Attribution License (https://creativecommons.org/licenses/by/4.0/). 

somata described in neonatal V1. While mentioning the existence of this fraction of PV neurons is essential, we do not believe they contribute significantly to the PFC. The paragraph below has been added to the discussion to reflect this (Ln 608-623).

"This might also, in part, reflect the expression of PV on a fraction of pyramid-shaped neurons reported across layer 5 in a range of mammals, including cats (Stichel et al. 1987), rats (van brederode, helliesen, and hendrickson 1991), marmosets (Spatz, Illing, and Weisenhorn 1994b), and mice (Tanahira et al. 2009; Peng et al. 2021). While the excitatory phenotype of some of the pyramid-shaped PV+ cells has been confirmed in rodents (Tanahira et al. 2009; Peng et al. 2021; van brederode, helliesen, and hendrickson 1991), their description in primates remains based on their morphology and in the primary visual cortex (V1; (Spatz, Illing, and Weisenhorn 1994b; Spatz, Illing, and Weisenhorn 1994a; Johnson and Casagrande 1995). This subpopulation, estimated to comprise 10~20% of the total PV+ population, was described in layer 5 of the neonatal marmoset V1 and appeared transient as it was absent in adult V1 (Spatz, Illing, and Weisenhorn 1994b). Our observations revealed that PV+ neurons in the postnatal PFC exhibited various morphologies, including some pyramid-like somata. However, their density was not as high as Spatz and colleagues observed in the neonatal V1. Therefore, whilst we cannot exclude that pyramid-shaped, potentially excitatory PV+ are present in the marmoset PFC, they can only form a negligible fraction compared to the typical PV+ interneurons"

**Reviewer 2:** The authors have improved the manuscript by adding data and rewriting the text. As previously stated, the strength of this study lies in the authors' first-time description of the developmental expression of interneuron markers in the PFC of marmosets. The gradual increase in Pvalb can be simply explained by the maturation of this cell type, and it is well-established that Pvalb is an activity-dependent gene in rodent circuits. The observed decline in Sst expression is intriguing; however, the current manuscript does not clearly distinguish whether this represents a reduction in Sst expression levels or a decrease in the number of Sst+ cells, despite addressing both protein and RNA levels of Sst. Reviewer 1 highlights the importance of demonstrating a marker for MGE-lineage interneurons, such as Lhx6, and suggests that if this is not possible, the authors should tone down the claim regarding a reduction in Sst+ cell numbers. I agree with this recommendation. A decrease in Sst interneurons during development is a significant claim, and I am not aware of similar observations in other cortical regions or animal models. If apoptosis (Figure S1) is not the cause, what mechanism explains the decrease in Sst+ cells? By utilizing MGE-lineage markers such as Lhx6 and Sox6, or even the pan-GABAergic marker Gad, the authors could clarify whether the observed decline reflects reduced Sst expression (in which case Lhx6 cell numbers would remain unchanged) or a decrease in Sst+ cell numbers (in which case Lhx6 levels would also decrease). Additionally, the manuscript would benefit from a plausible explanation for why Sst expression or Sst+ cell numbers decrease in the juvenile PFC of marmosets. Similarly to what I have stated in the original review, the revised version still lacks mechanistic insights to explain the observed decrease in Sst interneurons or the increase in the proteins of Pvalb and others during development. These issues should be addressed before the manuscript can be considered for publication in Development.

Exploring the underlying mechanism of the SST interneuron reduction during postnatal development requires a significant amount of experiment and work, which is out of the focus and intent of this descriptive study. Whilst our results demonstrate that the developmental reduction in SST+ neurons is not due to apoptosis, we cannot rule out other forms of cell death. For example, cells undergoing apoptosis-like programmed cell death exhibit less compact chromatin condensation and no caspase activation. Similarly, necrosis-like programmed cell occurs without chromatin condensation and caspase activation (for review, see (Kögel and Prehn 2013)). Unfortunately, these other forms of programmed cell death cannot be easily labeled with a simple antibody marker, like aCasp3 for apoptosis. Therefore, we cannot exclude that SST+ neurons undergo a caspase-independent programmed cell death. Alternatively, these neurons could down-regulate the expression of SST upon completing their roles, such as regulating the (Mòdol et al. 2024).

However, to improve the readability of the manuscript and further clarify the points made by Reviewer 2, the following has been added to the manuscript text for clarification: (Ln 530-563) "One likely reason for this observation may rely on the specialized functional contribution of interneuron subtypes in prefrontal circuits. It has been demonstrated that PV+ and SST+ subtypes

© 2025. Published by The Company of Biologists under the terms of the Creative Commons Attribution License (https://creativecommons.org/licenses/by/4.0/).

exhibit distinct tendencies to connect with long-range excitatory projections from various cortical areas (Naskar et al. 2021). Naskar et al. reported that PV+ cells in rodent primary somatosensory area (S1) receive predominantly long-range cortical inputs from other somatosensory areas to enable feed-forward inhibition of pyramidal neurons. In contrast, long-range cortical connections project weakly to the SST+ subtype. While these findings need to be examined in the PFC, the evidence suggests that prefrontal areas establish a dense network of cortical interconnectivity (Majka et al. 2016) to perform complex computations and enable higher cognitive functions. Therefore, it is plausible that the number of PV+ cells increases postnatally to support the maturation and regulation of complex prefrontal cortex computations. Interestingly, while there are apparent differences between primates and rodents, the upregulation of PV around adolescence has also been observed in the medial PFC of rats (Petros et al. 2015). This corroborates the suggestion that PV is essential for the refinement of prefrontal GABAergic function and connectivity, during which PV upregulation supports the acquisition of the mature GABAergic phenotype necessary to sustain adult PFC functions. The SST+ interneuron subtype is thought to receive relatively weak long-range cortical inputs (Naskar et al., 2021). Consequently, these cells may decrease in number or downregulate SST expression upon fulfilling their developmental roles, such as orchestrating the timing of cortical network desynchronization and facilitating the maturation of PV+ interneurons (Mòdol et al., 2024). However, the precise mechanism underlying the post-developmental reduction in SST+ cells—whether due to cell loss, although we suggest from our results, this is not apoptosis at the time points we analyzed or transcriptional downregulation of SST—remains to be elucidated" (p21/48).

Responses to the Original Major Points

1. It is reassuring to see that the decline in Sst expression is specific to the PFC and not observed in V1 (Figure S1). Thank you for this comment

2. The statement that "the developmental decline in Sst+ cells is not related to the development and maturation of PM thalamic connectivity networks with the PFC" does not provide mechanistic insights into why Sst+ cell numbers decrease during postnatal development. While I understand that manipulations in marmosets are challenging, if apoptosis (Figure S1) is ruled out, a reasonable explanation for this phenomenon remains necessary. Please see the response to the previous comment.

ADDITIONAL REFERENCES INCLUDED IN MANUSCRIPT:

Johnson, JK, and VA Casagrande. 1995. 'Distribution of calcium-binding proteins within the parallel visual pathways of a primate (Galago crassicaudatus)', Journal of Comparative Neurology, 356: 238-60.

Kögel, Donat, and Jochen HM Prehn. 2013. 'Caspase-independent cell death mechanisms.' in, Madame Curie Bioscience Database [Internet] (Landes Bioscience).

Majka, P., T. A. Chaplin, H. H. Yu, A. Tolpygo, P. P. Mitra, D. K. Wójcik, and M. G. Rosa. 2016. 'Towards a comprehensive atlas of cortical connections in a primate brain: Mapping tracer injection studies of the common marmoset into a reference digital template', J Comp Neurol, 524: 2161-81.

Mòdol, Laura, Monika Moissidis, Martijn Selten, Fazal Oozeer, and Oscar Marín. 2024. 'Somatostatin interneurons control the timing of developmental desynchronization in cortical networks', Neuron, 112: 2015-30.e5.

Mundinano, I. C., P. A. Flecknell, and J. A. Bourne. 2016. 'MRI-guided stereotaxic brain surgery in the infant and adult common marmoset', Nat Protoc, 11: 1299-308.

Naskar, Shovan, Jia Qi, Francisco Pereira, Charles R. Gerfen, and Soohyun Lee. 2021. 'Cell-type-specific recruitment of GABAergic interneurons in the primary somatosensory cortex by long-range inputs', Cell Reports, 34.

Peng, Hanchuan, Peng Xie, Lijuan Liu, Xiuli Kuang, Yimin Wang, Lei Qu, Hui Gong, Shengdian Jiang, Anan Li, Zongcai Ruan, Liya Ding, Zizhen Yao, Chao Chen, Mengya Chen, Tanya L. Daigle, Rachel Dalley, Zhangcan Ding, Yanjun Duan, Aaron Feiner, Ping He, Chris Hill, Karla E. Hirokawa, Guodong Hong, Lei Huang, Sara Kebede, Hsien-Chi Kuo, Rachael Larsen, Phil Lesnar, Longfei Li, Qi Li, Xiangning Li, Yaoyao Li, Yuanyuan Li, An Liu, Donghuan Lu, Stephanie Mok, Lydia Ng, Thuc Nghi Nguyen, Qiang Ouyang, Jintao Pan, Elise Shen, Yuanyuan Song, Susan M. Sunkin, Bosiljka Tasic, Matthew B. Veldman, Wayne Wakeman, Wan Wan, Peng Wang, Quanxin Wang, Tao Wang, Yaping Wang, Feng Xiong, Wei Xiong,

© 2025. Published by The Company of Biologists under the terms of the Creative Commons Attribution License (https://creativecommons.org/licenses/by/4.0/).

Wenjie Xu, Min Ye, Lulu Yin, Yang Yu, Jia Yuan, Jing Yuan, Zhixi Yun, Shaoqun Zeng, Shichen Zhang, Sujun Zhao, Zijun Zhao, Zhi Zhou, Z. Josh Huang, Luke Esposito, Michael J. Hawrylycz, Staci A. Sorensen, X. William Yang, Yefeng Zheng, Zhongze Gu, Wei Xie, Christof Koch, Qingming Luo, Julie A. Harris, Yun Wang, and Hongkui Zeng. 2021. 'Morphological diversity of single neurons in molecularly defined cell types', Nature, 598: 174-81.

Petros, Timothy J., Ronald S. Bultje, M. Elizabeth Ross, Gord Fishell, and Stewart A. Anderson. 2015. 'Apical versus Basal Neurogenesis Directs Cortical Interneuron Subclass Fate', Cell Reports, 13: 1090-95.

Preuss, Todd M., and Jon H. Kaas. 1996. 'Parvalbumin-like immunoreactivity of layer V pyramidal cells in the motor and somatosensory cortex of adult primates', Brain Research, 712: 353-57.

Spatz, W. B., R. B. Illing, and D. M. Weisenhorn. 1994a. 'Distribution of cytochrome oxidase and parvalbumin in the primary visual cortex of the adult and neonate monkey, Callithrix jacchus', J Comp Neurol, 339: 519-34.

Spatz, WB, R-B Illing, and DM Vogt Weisenhorn. 1994b. 'Distribution of cytochrome oxidase and parvalbumin in the primary visual cortex of the adult and neonate monkey, Callithrix jacchus', Journal of Comparative Neurology, 339: 519-34.

Stichel, C. C., W. Singer, C. W. Heizmann, and A. W. Norman. 1987. 'Immunohistochemical localization of calcium-binding proteins, parvalbumin and calbindin-D 28K, in the adult and developing visual cortex of cats: A light and electron microscopic study', Journal of Comparative Neurology, 262: 563-77.

Tanahira, Chiyoko, Shigeyoshi Higo, Keisuke Watanabe, Ryohei Tomioka, Satoe Ebihara, Takeshi Kaneko, and Nobuaki Tamamaki. 2009. 'Parvalbumin neurons in the forebrain as revealed by parvalbumin-Cre transgenic mice', Neuroscience research, 63: 213-23.

van brederode, J. F. M., M. K. helliesen, and A. E. hendrickson. 1991. 'Distribution of the calcium-binding proteins parvalbumin and calbindin-D28k in the sensorimotor cortex of the rat', Neuroscience, 44: 157-71.

---

Third decision letter

MS ID#: dev.204254R2

MS TITLE: Developmental dynamics of the prefrontal cortical SST and PV interneuron networks: Insights from the monkey highlight human-specific features

AUTHORS: Nafiseh Hosseini Fin; Adrian Yip; Leon Teo; Jack Scott; Jihane Homman-Ludiye; James Bourne
ARTICLE TYPE: Research Article

Dear Dr Bourne,

I am delighted to tell you that your manuscript has been accepted for publication in Development, pending our standard publication integrity checks.

© 2025. Published by The Company of Biologists under the terms of the Creative Commons Attribution License (https://creativecommons.org/licenses/by/4.0/).
